# Comprehensive gut microbiota composition and microbial interactions among the three age groups

**DOI:** 10.1371/journal.pone.0305583

**Published:** 2024-10-18

**Authors:** Jun Ma, Xiaohua Yang, Jianwu He

**Affiliations:** 1 School of Food and Biological Engineering, Shaanxi University of Science and Technology, Xi’an, Shaanxi, China; 2 Guangdong Provincial Key Laboratory of Microbial Safety and Health, State Key Laboratory of Applied Microbiology Southern China, Institute of Microbiology, Guangdong Academy of Sciences, Guangzhou, Guangdong, China; 3 Pulmonary and Critical Care Medicine, Tongchuan People’s Hospital, Tongchuan, Shaanxi, China; Jawaharlal Nehru Technological University Hyderabad, INDIA

## Abstract

There is a growing interest in studying the microbiota associated with aging by integrating multiple longevity researches while minimizing the influence of confounding factors. Here, we reprocessed metagenomic sequencing data from four different aging research studies and evaluated potential confounding factors in order to minimize the batch effect. Subsequently, we detected the diversity and abundance of the gut microbiome in three different age cohorts. Out of 1053 different bacteria species, only four showed substantial depletion across different age groups: *Ligilactobacillus ruminis*, *Turicibacter sp*. *H121*, *Blautia massiliensis*, and *Anaerostipes hadrus*. Archaea accumulated more in young individuals compared to elderly and centenarians. *Candida albicans* was more prevalent in centenarians, but *Nakaseomyces glabratus* (also known as *Candida glabrata*) was more common in elderly adults. *Shuimuvirus IME207* showed a significant increase in centenarians compared to both control groups. In addition, we utilized a Fisher’s exact test to investigate topological properties of differentially abundant microbiota in the co-occurrence network of each age group. Microbial signatures specific to different age stages were identified based on the condition: the reads showing differential abundance were higher compared to the other age groups. Lastly, we selected *Methanosarcina sp*. *Kolksee* for the Y group, *Prevotella copri* for the E group and *Shuimuvirus IME207* for the C group as representatives of age-related characteristics to study how their interactions change during the aging process. Our results provide crucial insights into the gut microbiome’s ecological dynamics in relation to the aging process.

## Introduction

Centenarians have a lower risk for aging-related illnesses and infectious diseases [1]. The combination of factors including genetics, and environmental elements related to longevity is not well understood [2]. The gut microbiota has been identified as a critical role in the relationship between sickness and health. For example, our earlier work demonstrated that antibiotic-resistance genes increase in the gut microbiota of elderly groups [3]. In recent times, the metagenomic data for longevity microbiota analysis has been applied to at least three aspects: bacteria profile and functional analysis [4–6]; entire microbiota community and functional analysis [2, 7–10]; comparison of the composition and function at taxonomic kingdom group [1, 11]. However, the majority of studies primarily focused on examining the link within the microbial domain during the aging process in one specific geographical area.

Batch effects are list of variation generated by technical factors or confounding biological variables, which can lead to false positive findings and hinder true signals of comprehensive analysis by integrating metagenomic datasets of multiple large-scale studies. On the one hand, the procedures, such as sequence depth per sample, used in metagenomics research are still significantly different [12]. Library size ranges of large studies frequently varies substantially among studies, which can effect the read assignment of the same microbe among samples. One the other hand, gut microbiota composition, comprising bacteria and other microbiota such as fungus, archaea, and viruses, is frequently altered by many factors such as food, location, health, or sickness [13].

In the gut microbiota community, microbes usually interact with each other, generating a strongly related ecological network [14]. Occurrence networks have been massively exploited in the ecological research aera for exploring characteristics of microbial interactions [15, 16]. Jing et al pointed out that molecular ecological networks (MENs) can be utilized to explore the potential microbial interaction and the influence of environmental stress and explain the aspects of microbial community structures [17]. However, several research groups have investigated the microbiome composition change during aging process and identified centenarian-related microbial signatures without considering the interactions between microbiome [7, 8, 18]. In this study, we re-analyze previously published gut metagenomes to investigate all age-related member of the microbial community and their interaction shifts across the three age groups.

## Materials and methods

### Ethics approval and consent to participate

These sequencing datasets were downloaded from public databases. The subjects involved in the database have obtained ethical approval. User can download relevant data for free for research and publish relevant articles. Our study is based on open-source data, so there are no ethical issues and other conflicts of interest.

### Batch effect removal

To integrate the metagenomic sequencing data from four studies, we performed batch effect removal and then filtered out samples that induced statistical differences in alpha diversity between studies based on the Kruskal-Wallis test and remained 270 centenarians (group C, 228 females and 42 males, aged 90 to 109 years), 177 elderly (group R, 91 females, and 86 males, aged 62 to 89 years), and 99 young adults (group W, 43 females and 56 males, aged 21 to 62 years). We manually curated metadata tables for the public cohorts, including sampleID, age, country, gender, run_ID, sequencing_platform, PMID, and numbers_reads. The sequencing data download See supplementary S1 Table for more information about cohorts.

### Metagenomics data analysis

To quality control of reads, the raw data of metagenomic sequencing was analyzed using Sunbeam v3.0.0 [19], including the removal of adaptors, low-quality, and low-complexity sequences by *Cutadapt*, *Trimmomatic*, and *Komplexity*. And the human-host-aligned reads were removed using *bwa*, implemented in *Sunbeam*. After the initial quality-control process, the remaining reads are classified taxonomically using *kraken2* v2.1.2 (—minimum-hit-groups 3) and *bracken* v2.7. Reads numbers for each species were normalized into a percentage of the total number of assigned reads per sample. Batch effects removal was performed with a recently developed R package *ConQuR* v2.0, which can remove batch effects by a composite non-parametric model correction while retaining real information for microbiome association analysis [26]. We evaluated the variability of the microbiome data induced by Batch and condition factors using PERMANOVA [20] R^2^.

### Statistical analysis

We performed all statistical analyses using the R software (v 4.2.1). Alpha and beta diversity analysis of taxa species among groups were calculated with R package *vegan* (v 2.6–4), and visualized using *ggplot2* (v 3.4.2) and *ggpubr* (v 0.6.0). We examined differences in multiple groups using a nonparametric Kruskal-Wallis test, followed by a two-tailed Wilcoxon’s rank sum test for the evaluation of differences for alpha diversity indices between two groups, and a P-value of < 0.05 was considered significant differences. Output data of each microbiome category were further analyzed with the *MaasLin2* function (multivariate association with linear models), a multivariate statistical framework, to identify significant differences in microbe abundance with the default settings (total-sum scaling, log-transform, LM). *P*-values were adjusted for multiple testing using a false discovery rate (FDR). The microbes between groups with adjusted *p-value* < 0.25 were considered age-related DAMs. The co-occurrence network of microbes was constructed with Pearson’s correlation (PEA) based on taxonomic abundances. The pair-wise PEA matric was calculated using the *rcorr* function in the R package *Hmisc* v 5.1–0. To ensure the robustness correlation of networks, only significant correlations (*P* < 0.05; |*r*| > 0.3) were retained for building the network. We used the *cluster_walktrap* function in the *igraph* v 1.4.3 package for topological analysis of the network, which can group closely related nodes. Over-representation analysis of the age-related DAMs in the cluster’s nodes using the combination of cluster’s nodes and age-related DAMs as background was performed with *runGSAhyper* function in R package *piano* v 1.12.0. the age-related DAMs significant enriched clusters were identified as aged-related clusters, visualized using *igraph*.

## Results

### Cohorts overview

With the development of sequencing technologies, large-scale, high-resolution human microbiome profile studies including hundreds to thousands of individuals have been enabled. We collected a total of 1.4 TB fastq data (about 16.4 million reads per sample) of 546 adult participants (age range: 21–109 years old) in Sardinia (Italy) [8], Sichuan(China) [10, 21], Japan [9] and Emilia Romagna region(Italy) [7], of whom 362 were women (Table 1). Wu et al.’s research recruited 19 long-living people (>99+ yr old), 23 elderly people (68–88 yr old) and 17 young people (21–33 yr old) from Sardinia, Italy, while the metagenomic sequencing data can be download from PRJEB25514. Zhang et al.’s study included total 95 Chinese who were living in Sichuan (China) and divided into the four groups: 28 healthy long-living people (91–103 yr old), 9 unhealthy long-living people (90–93 yr old), 31 elderly people (67–75 yr old) and 27 young people (24–48 yr old), and gut microbiome metagenomic sequencing was performed on the Illumina NovaSeq 6000 platform with PE150, while raw data of sequencing can be downloaded from PRJNA624763. The Japanese cohort comprised faecal samples of 176 long-living people (>100 yr old), 110 elderly people (85–89 yr old) and 44 young people (21–55 yr old) and the gut metagenome sequencing was performed on the Illumina NovaSeq 6000 platform with PE 150, while raw data of sequencing can be downloaded from PRJNA675598. Rampelli et al.’s study included subjects who were living in the *Emilia Romagna region*, *Italy*, aged 22–109 years with an average age of 85 years, and divided into 38 long-living people (>99 yr old), 13 elderly people (65–75 yr old) and 11 young people (22–48 yr old), and gut microbiome metagenomic sequencing was performed on the Illumina NextSeq PE150 platform, while sequencing data can be downloaded from PRJNA553191.

**Table 1:** Demographic details of samples.

			Age groups (yr)		
Cohort	City (Country)	Gender	Centenarians	Elderly	Young
PRJEB25514	Sardinia (Italy)	Male: 23	n = 19 (99–107)	n = 23 (68–88)	n = 17 (21–33)
		Female:36			
PRJNA624763	Sichuan (China)	Male: 39	n = 37 (90–103)	n = 31 (67–75)	n = 27 (24–48)
		Female: 56			
PRJNA675598	/(Japan)	Male: 104	n = 176 (100+)	n = 110 (85–89)	n = 44 (21–55)
		Female: 226			
PRJNA553191	Emilia Romagna region (Italy)	Male: 18	n = 38 (99–109)	n = 13 (65–75)	n = 11 (22–48)
		Female: 44			

### Batch effects removal

**Fig 1:**
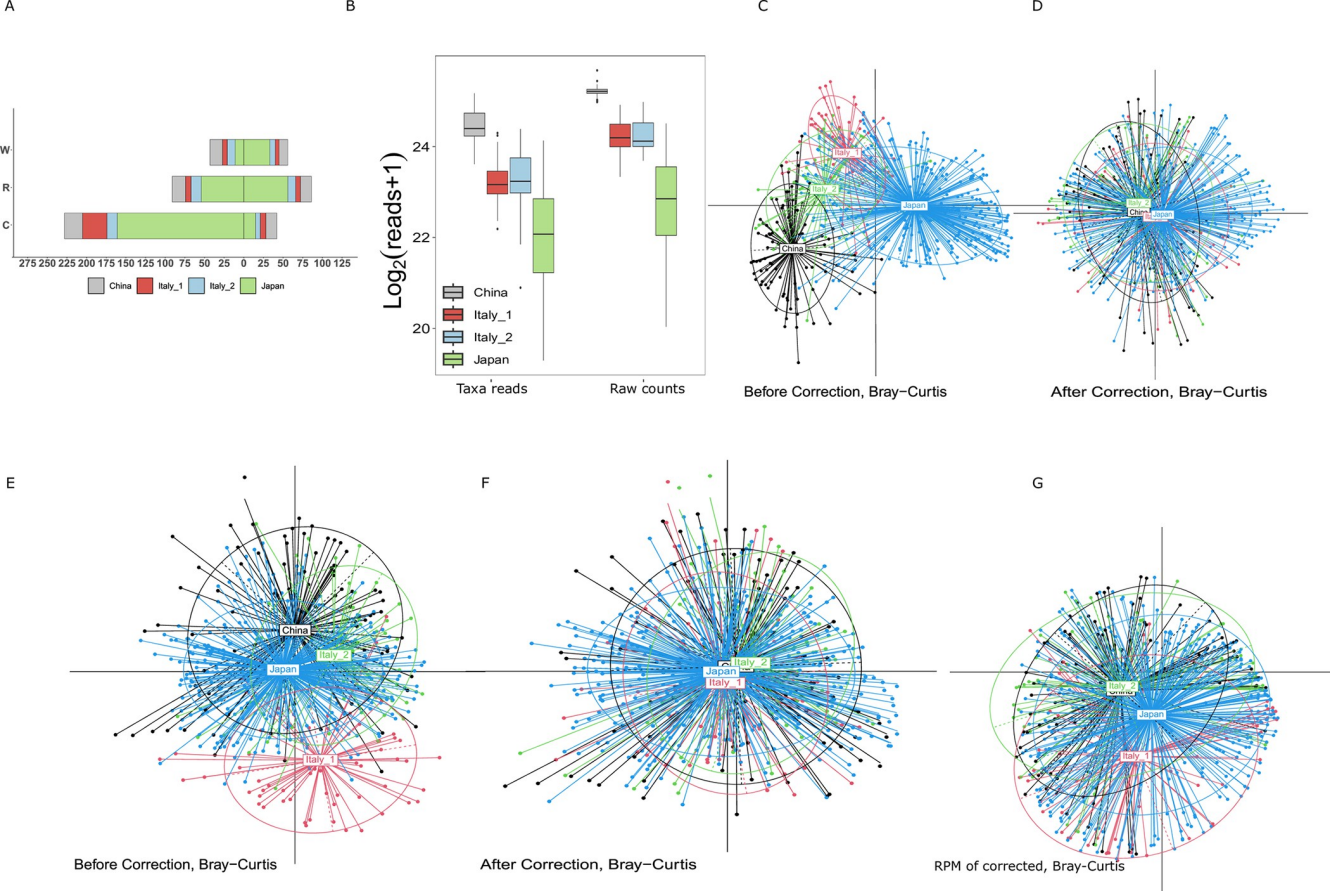
Batch effect removal. A. Gender distributions of the subjects among four cohorts within each age group. left side is female, right side is male. B Distribution of the number of assigned reads and sequenced reads in each dataset. C. D. E and F. PCoA plots clustered by batch ID according to Bray-Curtis dissimilarity on taxa reads, corrected taxa reads, normalized taxa reads, and corrected taxa read after normalization. G. PCoA plots clustered by batch ID based on Bray-Curtis dissimilarity on normalization data after corrected taxa reads. Each point represents a sample and each ellipse represents a batch, with the centroid indicating the mean. As an ellipse connects the 95% percentile of points for each batch, the size of the ellipse indicates the dispersion, and the angle indicates the higher-order features of the batch. Better alignment of the ellipses is preferred.

Sequence data with inter-study was frequently generated following differential handles and processing approaches, and many intra-studies include samples collected across times or locations and performed in different runs. These batch effects raise serious issues for meta-analysis and can lead to misleading results. Given the specific age of long-living individuals from Japan is missing, a total of 546 individuals were divided into three groups: group W (age < 62), group R (62≤age <89), and group C (age ≥89) (Fig 1A). According to the metadata in S1 Table, we chose age groups, gender, and raw counts as confounder factors to reduce the batch effects. The distribution of taxonomy reads and raw counts connected to each sample within cohorts varied (Fig 1B), while the associations between taxa read and raw counts are positively correlated based on the Pearson correlation value in each cohort, especially for the Japan cohort (S1 Fig). We observed that the substantial variations in data across the batches were largely reduced based on the mean (centroids) and dispersion (sizes of ellipses) after using the ConQuR processing (Fig 1C and 1D). Specifically, on the scale of normalized taxa reads (by Bray-Curtis dissimilarity), the means of four batches were centered to the same point, and the dispersion among batches is similar (Fig 1E and 1F). However, Fig 1G illustrates the normalization of corrected taxa read has not further reduced the batch impacts. Interestingly, the conditions (gender, age groups) with or without raw counts cannot modify the batch effects. The results of conditions merged with raw counts are the same as the original. The diversity in the taxa explained by batch and age condition was measured by ConQuR using PERMANOVA R2. As Table 2 shows, the correction of normalized taxa data substantially reduced the variability associated to batches and retained characteristic of condition compared with taxonomy reads.

**Table 2:** Clinical characteristics of each cohort.

PERMANOVA R^2^	Assigned reads		Relative abundance	
	(Bray-Curtis)		(Aitchison)	
	Batch	Age groups	Batch	Age groups
taxa read	0.1319	0.0408	0.1137	0.0201
ConQuR_taxa reads	0.0067	0.0602	0.0056	0.0259
RPM	0.0741	0.0502	0.0779	0.0246
ConQuR RPM	0.0025	0.0467	0.0043	0.0231
RPM after corrected taxa read	0.0058	0.0376	0.0040	0.0265

To investigate the taxonomy classification effected by the ConQuR approach, we compared the number of microbes identified within three age groups before and after read count adjustment. The overall number of microbes among age groups was decreased following batch effects elimination (S2A Fig). S2B, S2C Fig show ConQuR method has little influence on the proportion of bacteria, fungi archaea, and viruses in each age group. These results illustrate that the metagenome data generated from multiple research groups can be included into the meta-analysis after batch effect removal.

### Microbiome community alpha and beta diversity analysis in four batches

**Fig 2:**
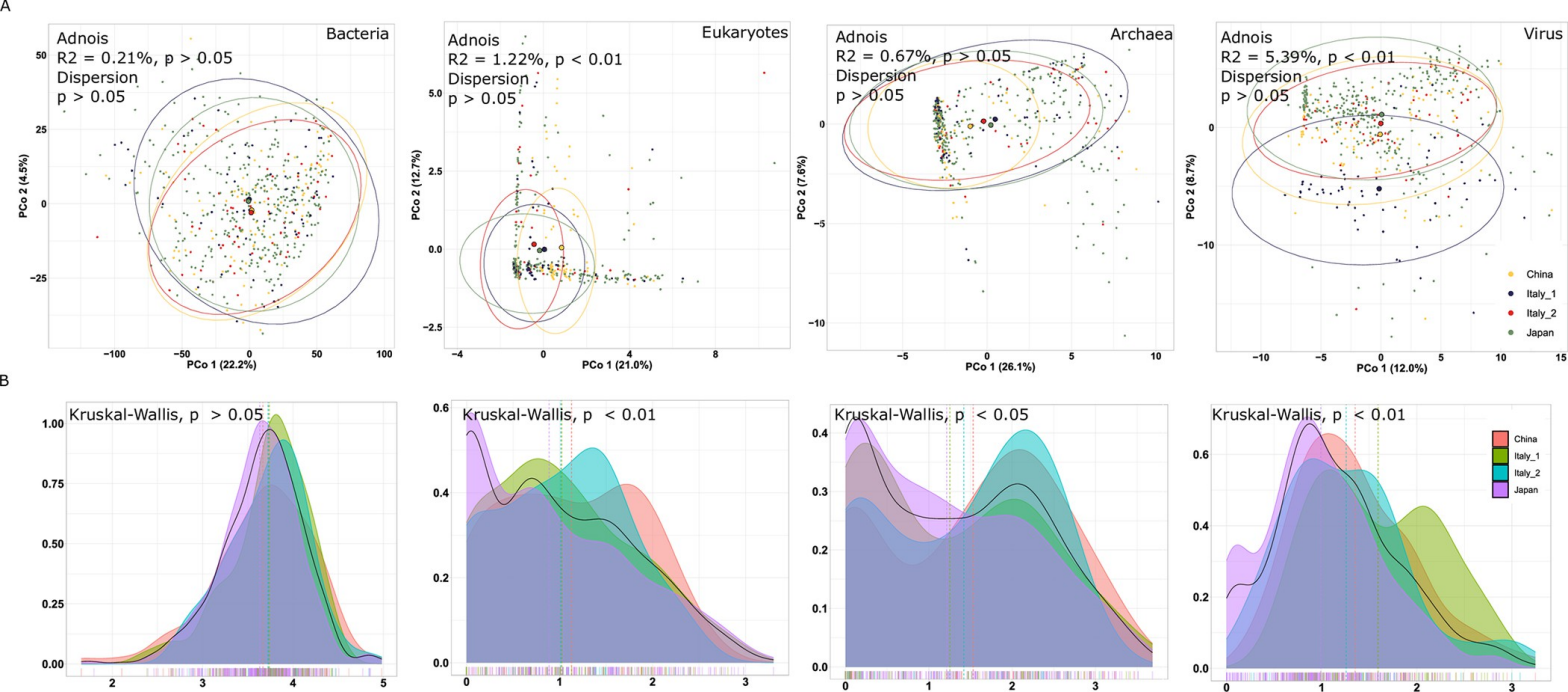
Microbiome community alpha and beta diversity analysis in four studies. A. Principal coordinate analysis (PCoA) of beta diversity (Bray-Curtis distance on the CLR-transformed) using bacterial, eukaryotic, archaeal and viral species. B. Shannon diversity index density analysis.

To further check the batch effect removal in gut microbial communities, a comparison of alpha and beta diversity for bacteria, eukaryotes, archaea, and viruses across locations was undertaken. Beta diversity of the samples’ microbial composition was measured depending on batches. Principal-coordinate analyses of the Bray-Curtis distance on the CLR-transformed microbiome species profiles were plotted to visualize the relatedness of the microbiota compositional profiles at the species level (Fig 2A). The clustering of the viral community was loosely clustered compared to bacterial, eukaryotic, and archaeal structures, where the communities were rather densely gathered. The Adonis and betadisper test showed that fewer effects of the variance in species profiles depend on batch IDs, even the fungal and viral communities were significantly clustered by individual samples among batches (*R*^*2*^ = 1.22%, *p* < 0.01; betadisper *p* > 0.05 and *R*^*2*^ = 5.39%, *p* < 0.01; betadisper *p* > 0.05). Additionally, to investigate whether subjects of each batch display different gut microbial communities, a comparison of alpha diversity for bacteria, eukaryotes, archaea, and viruses across cohorts was performed based on the Shannon diversity index, which accounts for species presence/absence and evenness (Fig 2B). There is no significant difference in the alpha diversity of gut bacteria among groups, meanwhile the bacteria had a higher alpha diversity than that of eukaryotes, archaea, and viruses, in which eukaryotes, archaea and viruses varied dramatically in different cohorts (Kruskal-Wallis test, *p* < 0.05).

### Microbiome community alpha diversity analysis in three age groups

**Fig 3:**
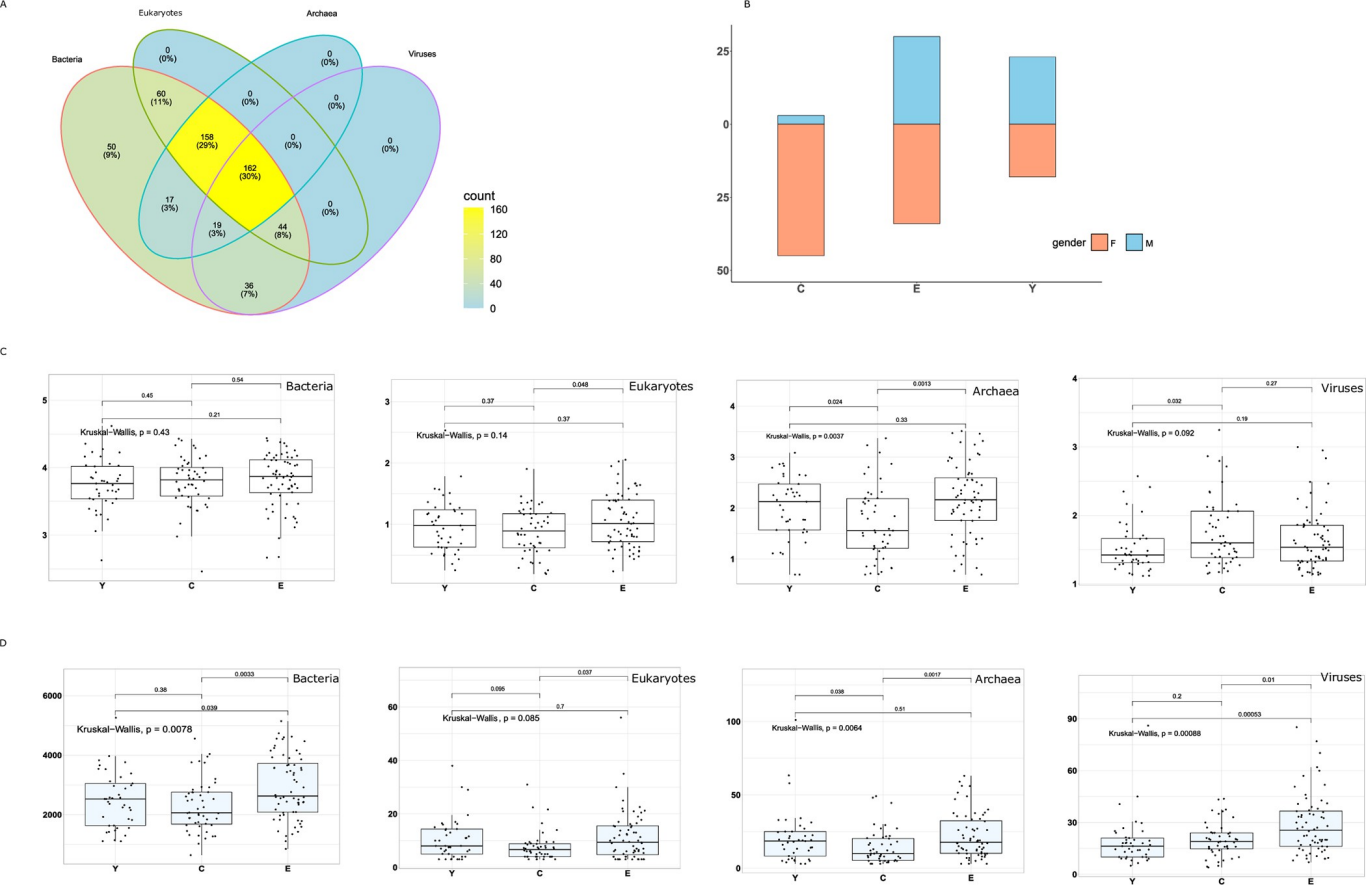
Significant differences in alpha diversity and richness analysis between the three age groups. A. The number of samples with no significant alpha diversity in each microbiota community is based on the Kruskal-Wallis test. B. The remaining subjects were divided into three groups depending on their age. Boxplot without colored means alpha diversity analysis (C) and boxplot filled with a color strand for richness analysis (D).

To integrate metagenomic sequencing of studies, we firstly filtered samples with statistically significant differences in alpha diversity between studies using the Kruskal-Wallis test (Fig 3A). The remaining subjects were categorized into three groups depending on their age: the long-living group C (age > 95; n = 48), the elderly group E (66<age <90, n = 64, median: 85 years old) and the young group Y (age <55, n = 41, median: 27 years old) (Fig 3B). The female in the centenarians is predominantly population that is widely documented in earlier longevity study [1, 7, 22]. We analyzed the alpha diversity and richness in three different age groups to gain a better understanding of how age influences microbial communities. There were no significant differences in Shannon index values among the three age groups of bacterial communities (Fig 3C). However, the Chao1 index was statistically indistinguishable between the C group and the E group, as well as between the Y group and the E group, based on the Kruskal-Wallis test followed by the Wilcoxon’s rank sum test (Fig 3D). For archaeal communities, the statistical differences of alpha diversity and richness in three age groups were presented. We found that the Shannon index of the fungal community in centenarians was statistically separated from the elderly groups, with the C group showing lower richness index compared to the other age groups. The Shannon index distribution for viruses was significantly lower in the young group compared to the centenarian group. However, no significant difference in richness analysis was observed in the young and centenarian groups. Interestingly, there was a notable variation in Chao1 richness between the E group and other groups. These results show that there are significant differences in centenarians compared to young and elderly individuals, as observed through alpha diversity and richness analysis.

### Microbiome community beta diversity analysis in three age groups

**Fig 4:**
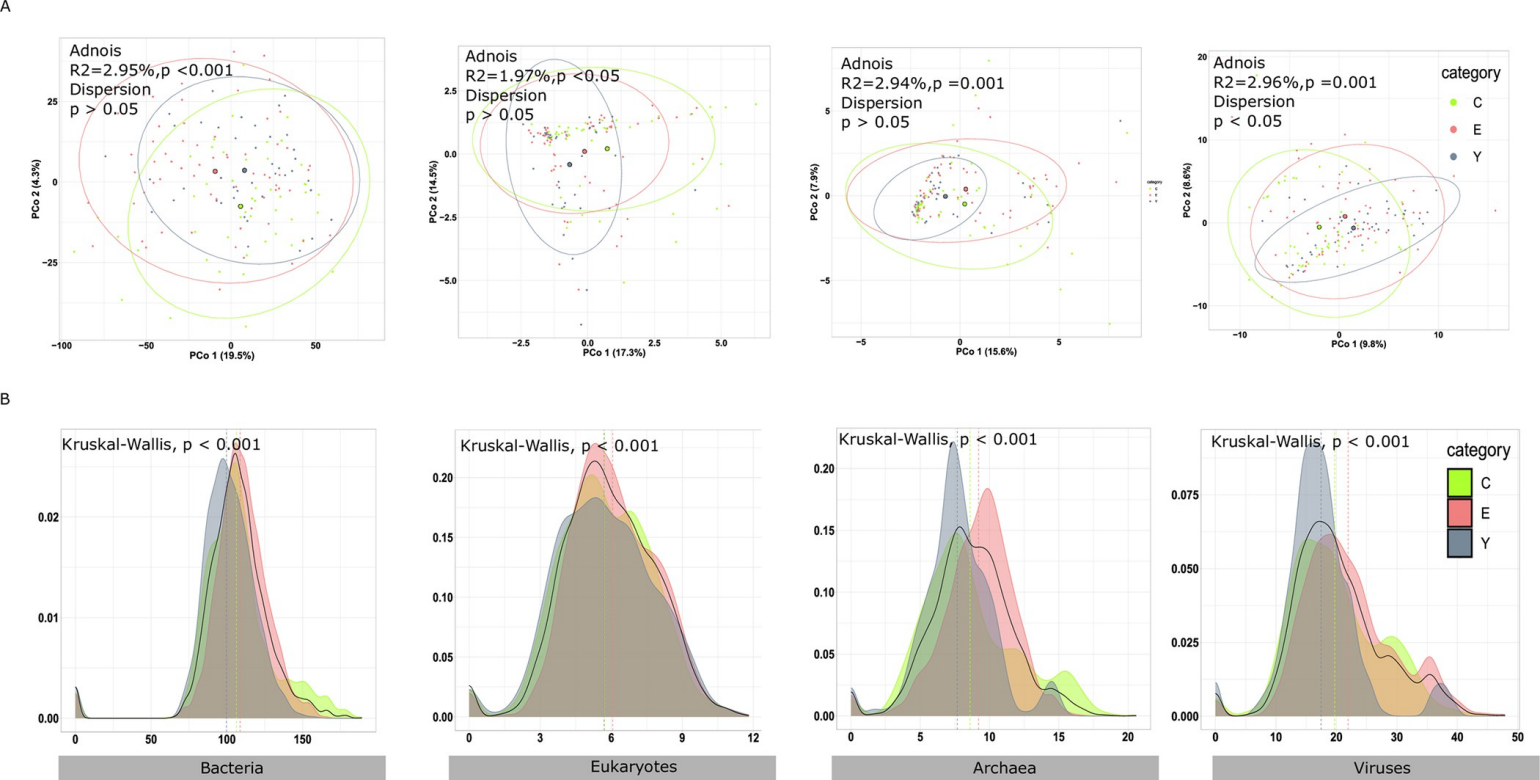
Bacterial, eukaryotic, archaeal, and viral community beta diversity in different age groups. A. PCoA of Bray-Curtis distances of the microbiota in each sample across the age group at the species level. B. Density plots of microbiota profile in community’s similarities analysis in each age group.

We measured the beta diversity of individual samples and divided them into groups depending on age categories. Principal-coordinate analyses of the Bray-Curtis distance were plotted to visualize the relatedness of the microbiota compositional profiles at the species level (Fig 4A). The clustering of the microbial communities was relatively loosely gathered. The *Adonis* test showed all microbial communities were statistically different between age groups, indicating microbiota profiles in each type of microbiota have higher differences in age groups. Additionally, the structural differences of bacterial, fungal, archaeal, and viral communities among individual samples were assessed to evaluate the effects of age categories (Fig 4B). The distribution of distances demonstrated the varied extents of microbial community structures divided by age categories, which are dissimilar based on the Kruskal-Wallis test (*P* < 0.001). The highest point degree of distance in the four communities are E group compared with that of the Y and C groups.

### Taxonomic compositions of microbiota communities

**Fig 5:**
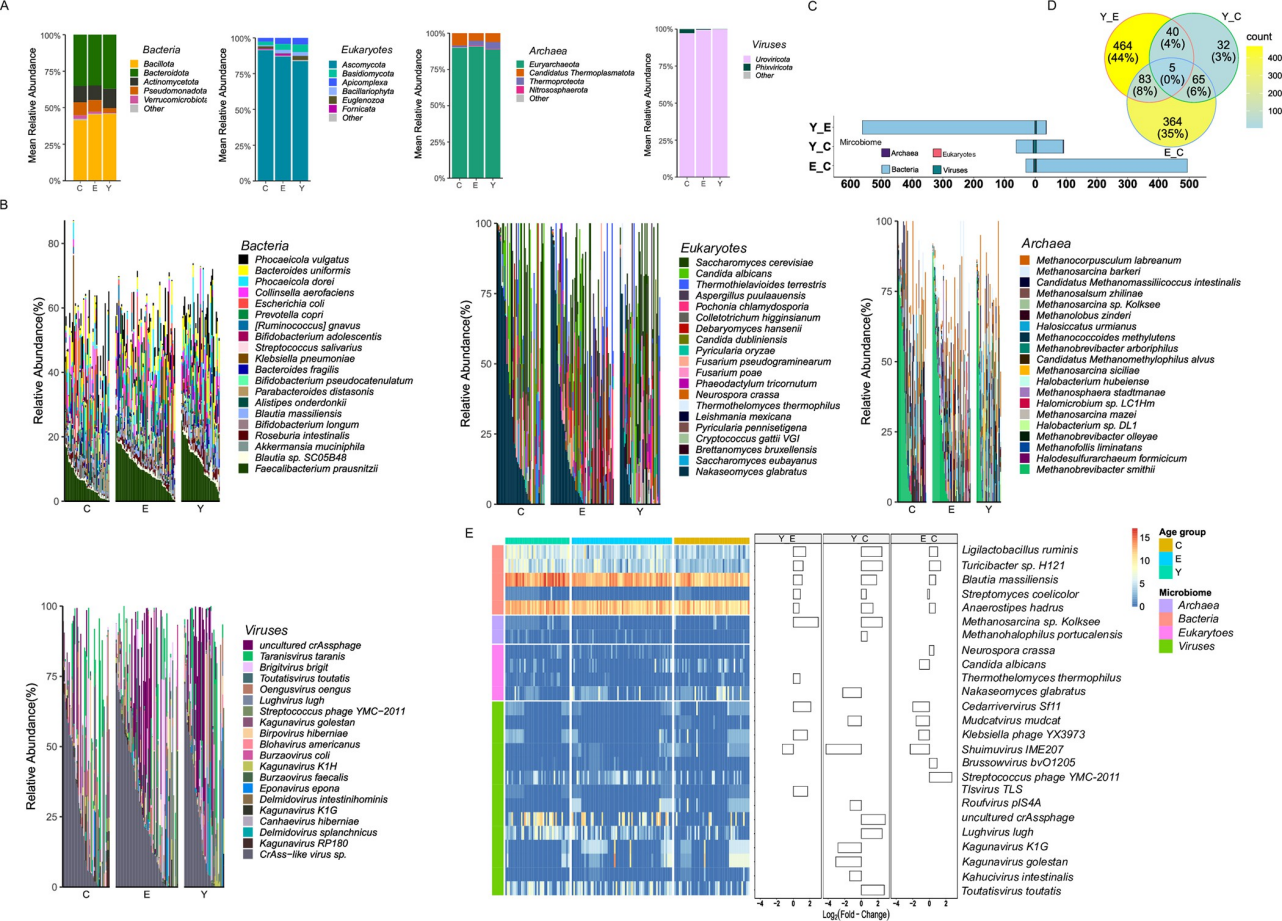
Taxonomic composition of microbiota community in the three age groups. A The plots show the mean relative abundance of different dominant bacterial, eukaryotic, archaeal and viral phylum across samples in each age group. B Relative abundance of majority enteric microbiota species in three age groups. C The number of differentially abundant microbiota between young, younger elderly, and centenarian groups. D The Venn diagram shows five different bacterial species present in three comparison groups. E Heatmap showing the log_2_(reads+1) of selected microbiota among samples and bar plot illustrates the log_2_(Fold change) value of corresponding microbiota in each comparison group.

Among the bacterial taxa, we observed the phylum Bacillota, Bacteroidota and Actinomycetota were dominant microbiota among three age groups, which is consistent with previous centenarian studies [9]. Ascomycota and Basidiomycota were the major fungal phyla in the enteric microbiome under the three age groups. The dominant archaea were Euryarchaeota and Candidatus Thermoplasmatota, while the primary viruses in the three groups were Uroviricota and Phixviricota (Fig 5A). We then focus on the relative abundance of microbiota at the species level. *Faecalibacterium prausnitzii* and *Phocaeicola* vulgatus are the most abundant bacteria, with 11.8% and 5.19% relative abundance, respectively. The main abundant eukaryotic species are *Nakaseomyces glabratus*, *Saccharomyces cerevisiae* and *Candida albicans*, occupying 25.8%, 20% and 12.7% relative abundance, respectively. Archaea orders with greater than 5% in the three age groups included *Methanobrevibacter smithii*, *Methanocorpusculum labreanum*, *Methanosarcina barkeri*, *Methanosarcina sp*. *Kolksee* and *Candidatus Methanomassiliicoccus intestinalis*, while the dominant viruses were *CrAss-like virus sp*. and *uncultured crAssphage*, occupied greater than 42% relative abundance of the total enteric viruses (Fig 5B).

To obtain a different abundant microbiome, a species-level relative abundance comparison was undertaken across the three age groups. The results showed that 1053 bacterial species, 4 eukaryotic species, 2 archaeal species and 14 viral species had differential abundance in the three age groups. The large number of microbes in the elderly age group showed substantial differences in abundance among young and centenarians compared with that identified by young versus centenarians (Fig 5C). The majority of differential abundant bacterial groups identified by three comparison groups including young vs. elderly, young vs. centenarians, and elderly vs. centenarians were group-specific, only 5 organisms (*Ligilactobacillus ruminis*, *Turicibacter sp*. *H121*, *Blautia massiliensis*, *Streptomyces coelicolor* and *Anaerostipes hadrus*) of them were commonly present (Fig 5D). Moreover, we showed the abundance of five common bacteria and all differential abundant eukaryotes, archaea and viruses within a heatmap. The bar plot in Fig 5E presents the enrichment and depletion of the respective microbes in the young group compared with the elderly and centenarian, and in the elderly group compared with centenarians. The five common differential abundant bacteria species were gradually depleting from young to centenarians, except *Streptomyces coelicolor* which enriched in centenarians. Archaea showed accumulation in the young compared with the elderly and centenarians. For example, *Methanosarcina sp*. *Kolksee* was enriched for the young. *Candida albicans* was enriched in centenarian individuals whereas *Nakaseomyces glabratus* was more accumulated in elderly groups. For viruses, we observed a large enrichment of *Shuimuvirus IME207*, a moderate enrichment of *Cedarrivervirus Sf11* and a depletion of *Streptococcus phage YMC-2011* in centenarians compared to both control groups. These results indicate the enteric microbiome composition shifting is in part related to the aging process in individuals of extraordinary longevity.

### Network analysis for exploring the microbiome interactions shift

We then performed additional microbiome differential abundance analysis and created the ecological network at the species level to explore the potential interplay of differentially abundance microbes during the aging process. Co-occurrence networks are a prominent method used in microbial ecology for inferring the association between samples of microbial communities based on taxonomic composition data. The co-occurrence network of young gained a total of 787543 edges among 6986 nodes and was split into 26 large microbiota clusters (membership > 10). There were 7550 nodes with 2212233 edges in the elderly network, which generated 24 large microbial communities. Meanwhile, we identified 27 large microbial clusters in the centenarian network including 7135 nodes with 2087937 edges.

**Fig 6:**
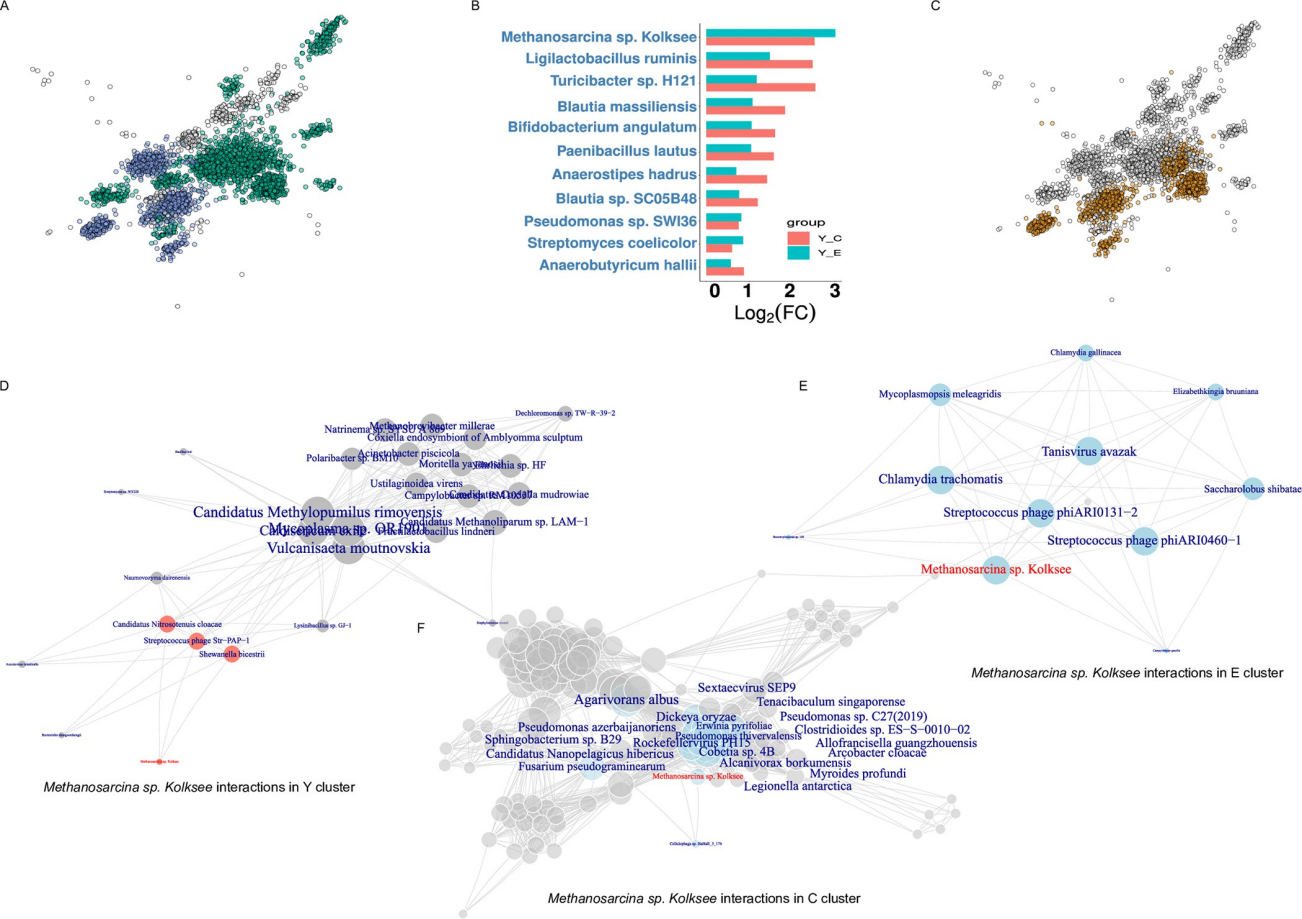
Age-related microbial biomarker interaction shift. A Differential abundance microbiota of Y vs. E, and Y vs. C significant enriched cluster. Common enriched clusters in blue, green color indicate differential abundance microbiota specific for Y vs. E. B Young group-related microbial biomarkers. C Clusters containing age-related microbial biomarkers in yellow. D, E and F show *Methanosarcina sp*. *Kolksee* interactions in the clusters of Y, E, and C networks, respectively.

To investigate the topological properties of differential abundance microbiota in each co-occurrence network, we independently assess whether the differential abundance microbiota significantly enriched in major microbial clusters using Fisher’s exact test. We observed that differential abundance species generated by young vs. centenarian and young vs. elderly comparisons were separately enriched in 5 common clusters of the young network, while the rest of the differential abundance organisms in the young compared to the elderly were enriched in 13 clusters (Fig 6A). In elderly and centenarian networks, we also found the majority of clusters enriched by differential abundance species produced by elderly and centenarian group-related comparisons (E vs. Y, E vs C and C vs. Y, C vs. E) were generally (S3 and S4 Figs). The nodes of each common cluster were in blue. These findings show that the differential abundance species of distinct comparison groups may have close ecological associations within the gut microbial community.

Several species showed differential abundances in comparison groups, which we categorized into age stage-related signatures based on the condition: species abundance was higher compared with that in the remainder of the age groups. Eventually, we obtained 11 Y signatures, 83 E signatures and 13 C signatures (Fig 6B, S5 and S6 Figs). As pervious described, we also detected ecological clusters in their related network using signatures enrichment analysis and marked these nodes in yellow (Fig 6C, S7 and S8 Figs). We further investigated the life stage signatures interaction shift during the aging process by selecting three signatures from each age group including *Methanosarcina sp*. *Kolksee* for the Y group, Prevotella copri for the E group and *Shuimuvirus IME207* for the C group as examples. *Methanosarcina sp*. *Kolksee*, energy producer [23], were higher abundance species in the Y group compared with the E group and C group, whereas fewer interaction with microbes in the cluster of Y network compared with that in the cluster of E and C networks, respectively (Fig 6D–6F). We observed that 83 microbes interacted with *Prevotella copri* in the E cluster, 124 interactions in the Y cluster and 14 interactions in the C cluster (S9–S11 Figs). Bacteriophage *Shuimuvirus IME207*, which can lyse *Klebsiella pneumonia* and *Salmonella* [24], interacted with 26 viruses, 2 archaea and 8 bacteria in the C group cluster, and connected with more various microbiota including 1 archaea, 18 bacteria, 2 eukaryote and 20 viruses in E network (S12 and S13 Figs). However, IME107 was not present in the Y network. These results indicate that the interaction of age-related microbial marker change is independent of their abundance.

## Discussion

The human enteric microbiome has long been believed as a pivotal player in determining the health condition of aging adults. In this study, we collected four metagenome datasets across different locations and reassigned these datasets into the three age groups after batch effect removal. We found that the microbiome profiles in the elderly groups were more complex than that in the young and long-living groups, with bacteria token a substantial proportion of the number of identified gut microbiomes, followed by archaea, viruses and eukaryotes. Furthermore, the dominant species of each taxonomic type usually were prevalent among the three age groups. The overlapping of differential abundance microbiota within both comparative age groups is less, however these differential abundance microbiotas are independently enriched in the common clusters of an age group network. Finally, we observed that the interaction of microbiome signatures for each age group was dramatically changed depending on the ecosystem clusters during the aging process. More robust batch effect correction method is needed when integrating data from multiple sources and the variables are usually interdepend and multifacted contributing to batch effects. Unlike the transcriptome data, the fundamental properties of microbiome data are frequently extremely zero-inflation, over-dispersion, and diverse with complex distributions. Many batch effect correction algorithms, including as Limma, DESeq2, and ComBat, were originally developed for RNA-seq datasets and may not be totally suitable for metagenomic data analysis [25]. Secondly, confounding biological variables including geography, gender, BMI, age, stress, genetic, demographic, clinical and diet also lead batch effects to the microbiota composition analysis. A detailed assessment of the batch variables to batch effect correction is crucial. However, ComBat, percentile normalization, multivariate RUVIII and related methods try to eliminate batch effects from the raw data to generate corrected data without considering the interaction between batch effects and confounders of interest. To address these difficulties, multiple approaches have been developed for metagenomic data with different strategies: PLSDA-batch, BDMMA, NetMoss and ConQuR utilize Partial Least Squares Discriminant Analysis, Dirichlet-multinomial regression model and adopt the Bayesian framework, microbial network modules and Non-parametric modeling with two-part quantile regression model, respectively [25–28]. However, a promising method for the future of batch effect correction analysis prior to downstream analysis of metagenomic data are still need to be decided.

In addition to the raw count normalization before batch effect correction, removing library size different before removal batch effect was also proposed as an essential step to reduce the batch effect. We checked each fastq file with fastqc software and found that the median of read depth per sample across four cohorts are PRJEB25514: 18M, PRJNA624763: 38M, PRJNA675598: 0.7M, PRJNA553191:19M, respectively. In agreement with the previous study [26], combining the ConQuR and raw count of taxa transformed to relative abundance can dramatically reduce the batch effect, compared with the results generated by one of these two methods. However, an alternative ConQuR-libsize function, separately considering library size as a cofactor during batch removal, was not removing batch effects in this study, which is not consistent with the description in the ConQuR article. This may be caused by library size variation is not a substantial difference between batches.

To better understand the inconsistent results, additional factors influence should be removed prior to comparison results of the microbiome data analysis with previous works. Even though we found that bacterial, eukaryotic and archaeal communities in the elderly illustrated greater alpha diversity and beta diversity than that of the young and centenarians, in agreement with previous articles [4, 11], whereas in contrast with Wang et al reported [6]. And viral community in the centenarians exhibited a higher Shannon index than did young and elderly, in disagreement with previous literature [1]. It is hardly to determent the ground truth of age-related microbiome community diversity among previous studies is agreement or not. The inconsistent results were possibly caused by additional factors, such as the selection of software and database for metagenomics sequence analysis, or age thresholds for assigning samples into the three age groups: young, elderly and centenarians. For example, Xu et al. selected individuals aged from 94 to 105 as centenarians and picked ages from 50 to 59 as a threshold for the elderly [4]. Li et al. selected individuals with age from 100 to 106 as centenarians, elderly with age 66 to 92 [2]. Lu used age range from 99 to 107, 68 to 88, 21 to 33 to divide samples into centenarians, elderly and young groups [8].

The tendency of taxonomic abundance shift across the three age groups cannot provide enough evidence for age-dependent microbiome identification. The abundance of dominant species might have fewer substantial differences between age groups. For instance, *Faecalibacterium prausnitzii*, *Nakaseomyces glabratus*, *Methanobrevibacter smithii*, and *CrAss-like virus sp*. were the major bacteria, fungi, archaea and virus across three age groups. We observed that the abundance of *Faeacalibacterium prausnitzii*, a key butyrate producer, was decreased with aging which is consistent with what Biagi et al reported [7, 9, 22], whereas no significant difference between the young group and the elderly group. The pathogen *N*. *glabratus* [29], known as *Candida glabrata*, was enriched in centenarian and elderly groups, with no significant difference between the elderly and young group, and between centenarian and elderly groups. Furthermore, previous articles mentioned that the relative abundance of *M*. *smithii* was increased in long-living people [9]. However, we observed that the methanogen *M*. *smithii* was not enriched in centenarians but also in elderly groups, indicating the enrichment of this methanogen may start from an earlier stage of the aging process. Human digestion contains three steps: hydrolysis, fermentation and methanogenesis. Methanogenesis perform a significant part for enhancing the generation of ATP and short-chain fatty acids by consuming formate and hydrogen, which benefit for energy harvest for the host [30]. Compared with acetogens, *M*. *smithii* with the very low H_2_-utilization capacity can more efficiently deplete H_2_ from gut environment [31]. CrAss-like phages [32] are highly abundant across the three age groups with less difference between any pair of age groups.

Majority of co-occurrence network algorithms scarely reflect all interactions of pair species in real habitats. The network building approach usually infers an ecological association between interpreting the species interactions. Multiple algorithms have been developed for building microbial ecological networks. The classical methods are Pearson’s and Spearman’s correlation coefficient [33]. Sparse Correlations for Compositional data utilize an iterative approximation strategy to calculate the correlation between the log-ratio transformation abundances of the microbiome [34]. SParse InversE Covariance Estimation for Ecological ASsociation Inference (SPIEC-EASI) is another method used to infer species interactions network [35]. Hirano et al evaluated compositional-data approaches with realistic simulations and pointed out that the SPIEC-EASI and the SparCC are more challenging to infer microbial ecological networks than traditional methods [36]. To exclude the erroneous interactions, unlike previous studies that largely focused on the degree of species decided by interactions of species in the network, we selected the links between age-related biomarkers within the specific cluster. However, our study has some limitations that need attention. Firstly, this study needs for future analyses on a larger number of participants and on better-age classification to draw more reliable conclusions. Secondly, the to better understand the potential mechanisms of age-related microbiota variations, avoid false positive microbes, more accurately detect ecological interaction of pair species [37], we still need to compare the preformation of alternative metagenomic analysis software such as MetaPhlAn [38], CLARK [39]. Thirdly, given network-building methods inferring interaction based on parametric statistical models, alternative methods are also needed.

In summary, we presented a comprehensive analysis of all microbiome composition profiles across the three age groups, identified age-related microbiomes based on the difference in taxonomic abundance and interpreted the age-dependent microbes association shift across aging.

## Supporting information

S1 Table Metadata of four cohorts. (XLSX)

S1 Fig Association between raw counts and assigned reads among location samples. Pearson correlation coefficient(PCC) of each dataset, PCCJapan = 0.96, PCCItaly_1 = 0.69, PCCItaly_2 = 0.77, PCCChina = 0.34.
(PDF)

S2 Fig Number of taxa in three age groups. The total number of microbes among age groups was decreased after batch effects removal. B The proportion of bacteria, eukaryotes, archaea, and viruses in each age group before correction. C The proportion of bacteria, eukaryote, archaea and viruses in each age group after correction.
(PDF)

S3 Fig The clusters enriched by differential abundance species produced by E vs. Y, E vs C group comparisons. (PNG)

S4 Fig The clusters enriched by differential abundance species produced by C vs. Y and C vs. E group comparisons. (PNG)

S5 Fig Elderly group-related microbial biomarkers. (PDF)

S6 Fig Centenarian group-related microbial biomarkers. (PDF)

S7 Fig The ecological clusters in the elderly network using signatures enrichment analysis and marked these nodes in yellow. (PNG)

S8 Fig The ecological clusters in the centenarian network using signatures enrichment analysis and marked these nodes in yellow. (PNG)

S9 Fig 83 microbes interacted with *Prevotella copri* in the E cluster. (PDF)

S10 Fig 124 microbes interacted with *Prevotella copri* in the Y cluster. (PDF)

S11 Fig 14 microbes interacted with Prevotella copri in the C cluster. (PDF)

S12 Fig Bacteriophage *Shuimuvirus IME207* interacted with 26 viruses, 2 archaea and 8 bacteria in the C group cluster. (PDF)

S13 Fig Bacteriophage *Shuimuvirus IME207* connected with more various microbiota including 1 archaea, 18 bacteria, 2 eukaryote and 20 viruses in E network. (PDF)
