# Supplementary figures and images for: Comprehensive gut microbiota composition and microbial interactions among the three age groups
Source: PLoS One. 2024 Oct 18;19(10):e0305583. doi: 10.1371/journal.pone.0305583 (PMC11488730; doi:10.1371/journal.pone.0305583)

**Japan**

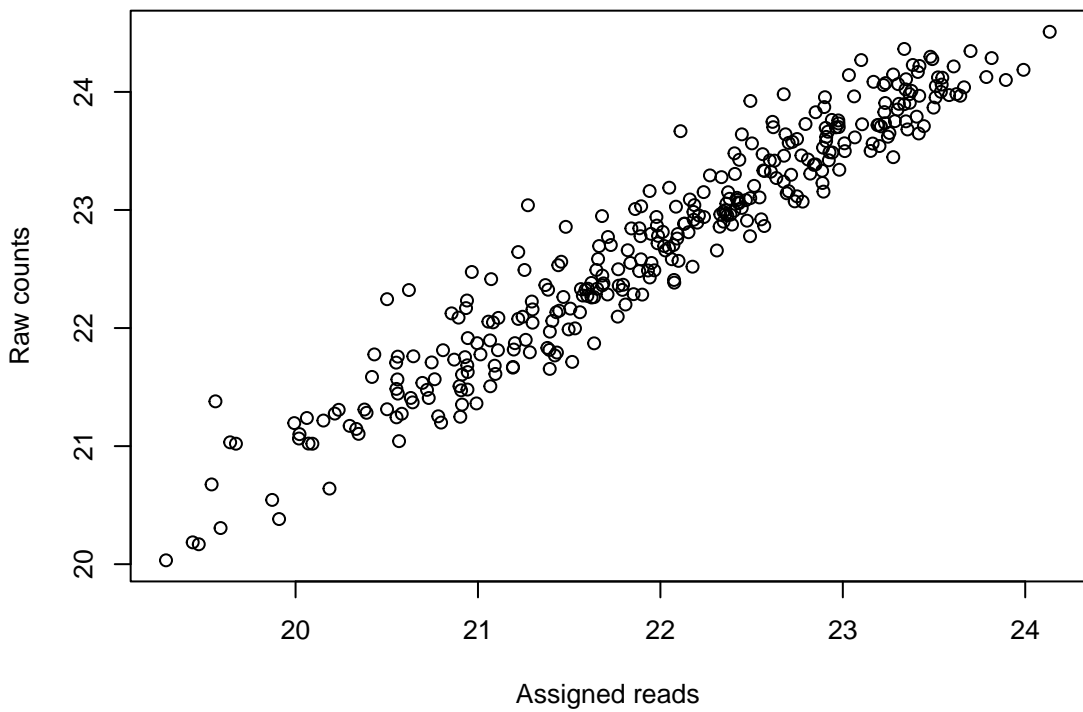

**Italy\_1**

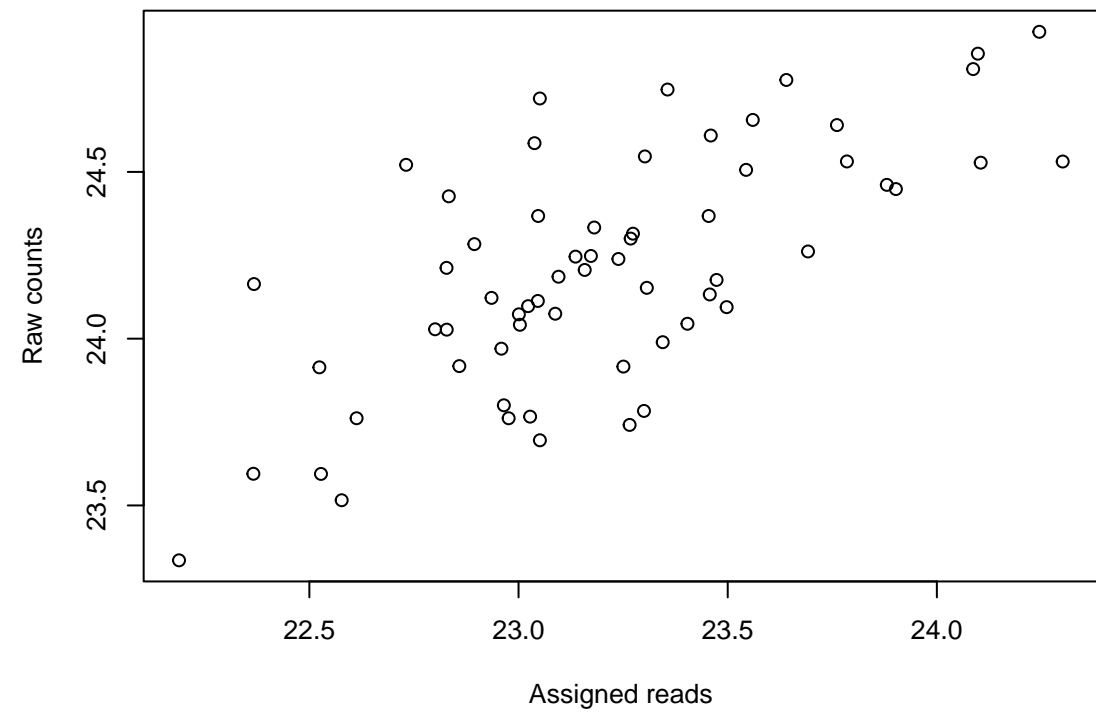

**Italy\_2**

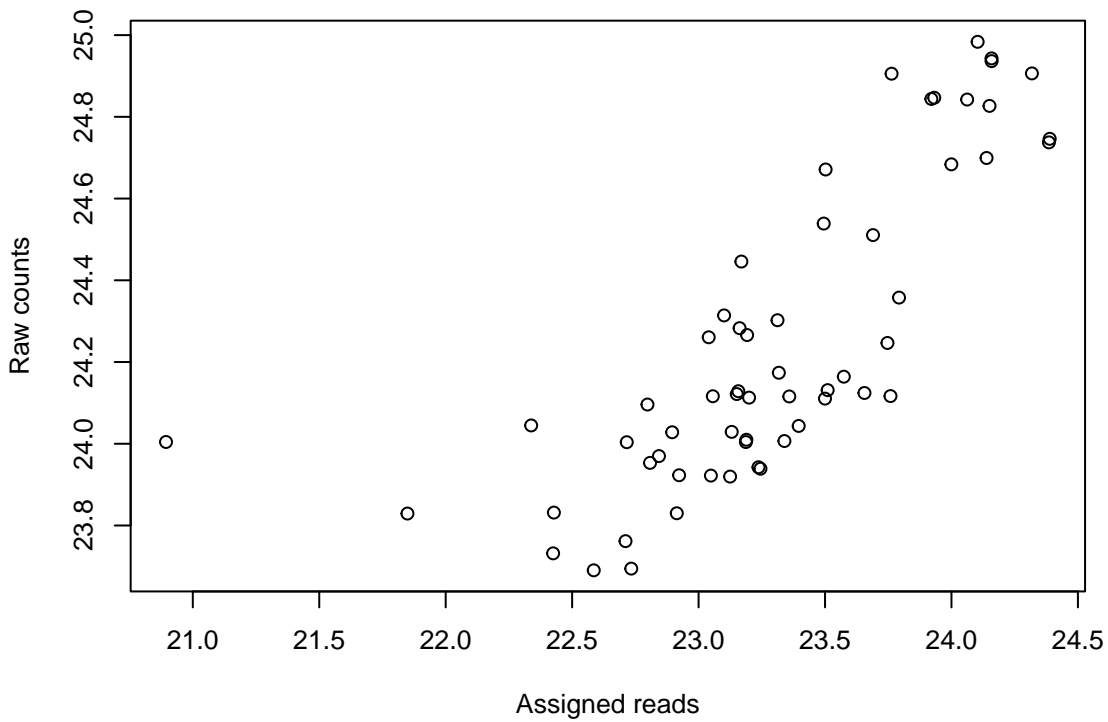

**China**

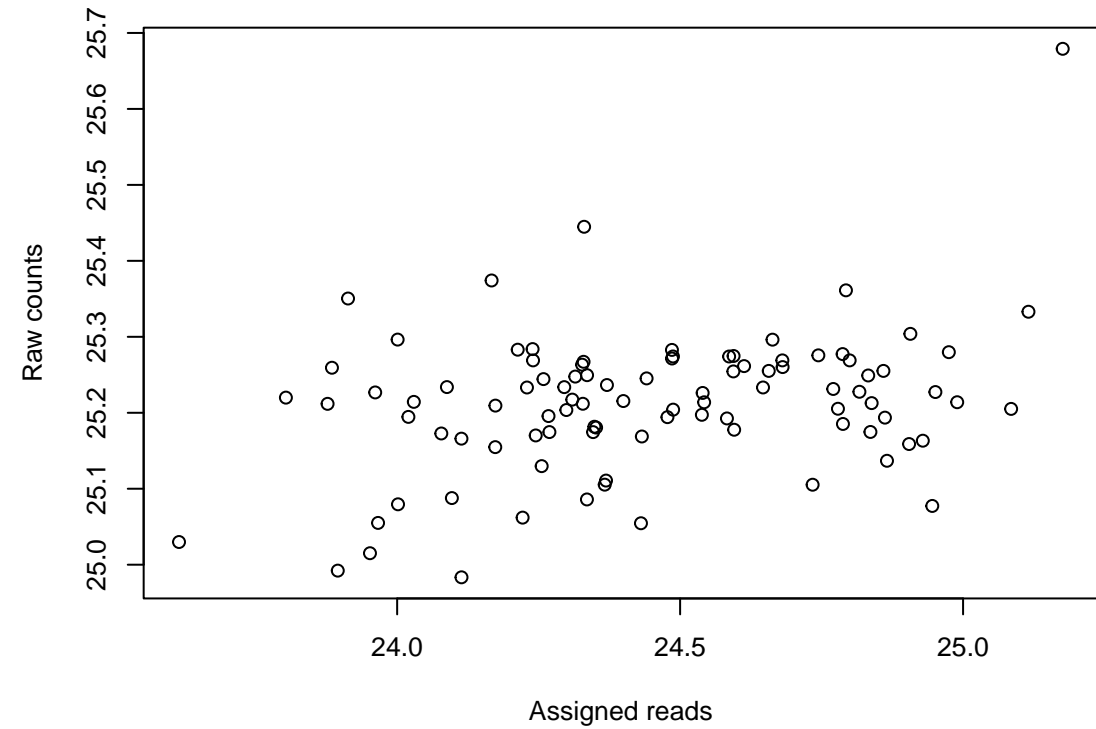

Supplement: S1 Fig — Pearson correlation coefficient(PCC) of each dataset, PCCJapan = 0.96, PCCItaly_1 = 0.69, PCCItaly_2 = 0.77, PCCChina = 0.34. (PDF) [file pone.0305583.s002.pdf]

A

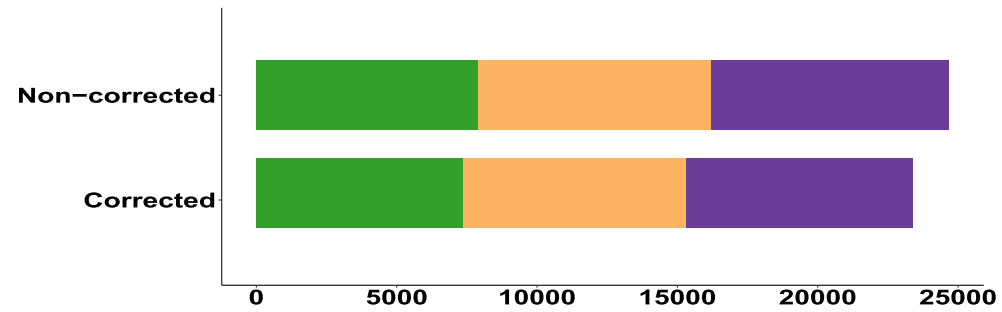

B

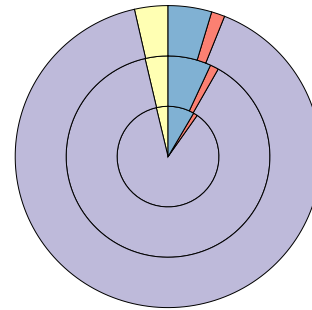

C

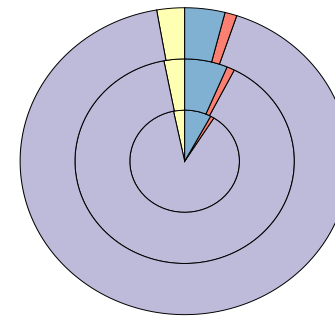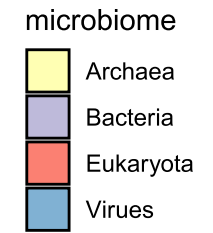

Supplement: S2 Fig — The total number of microbes among age groups was decreased after batch effects removal. B The proportion of bacteria, eukaryotes, archaea, and viruses in each age group before correction. C The proportion of bacteria, eukaryote, archaea and viruses in each age group after correction. (PDF) [file pone.0305583.s003.pdf]

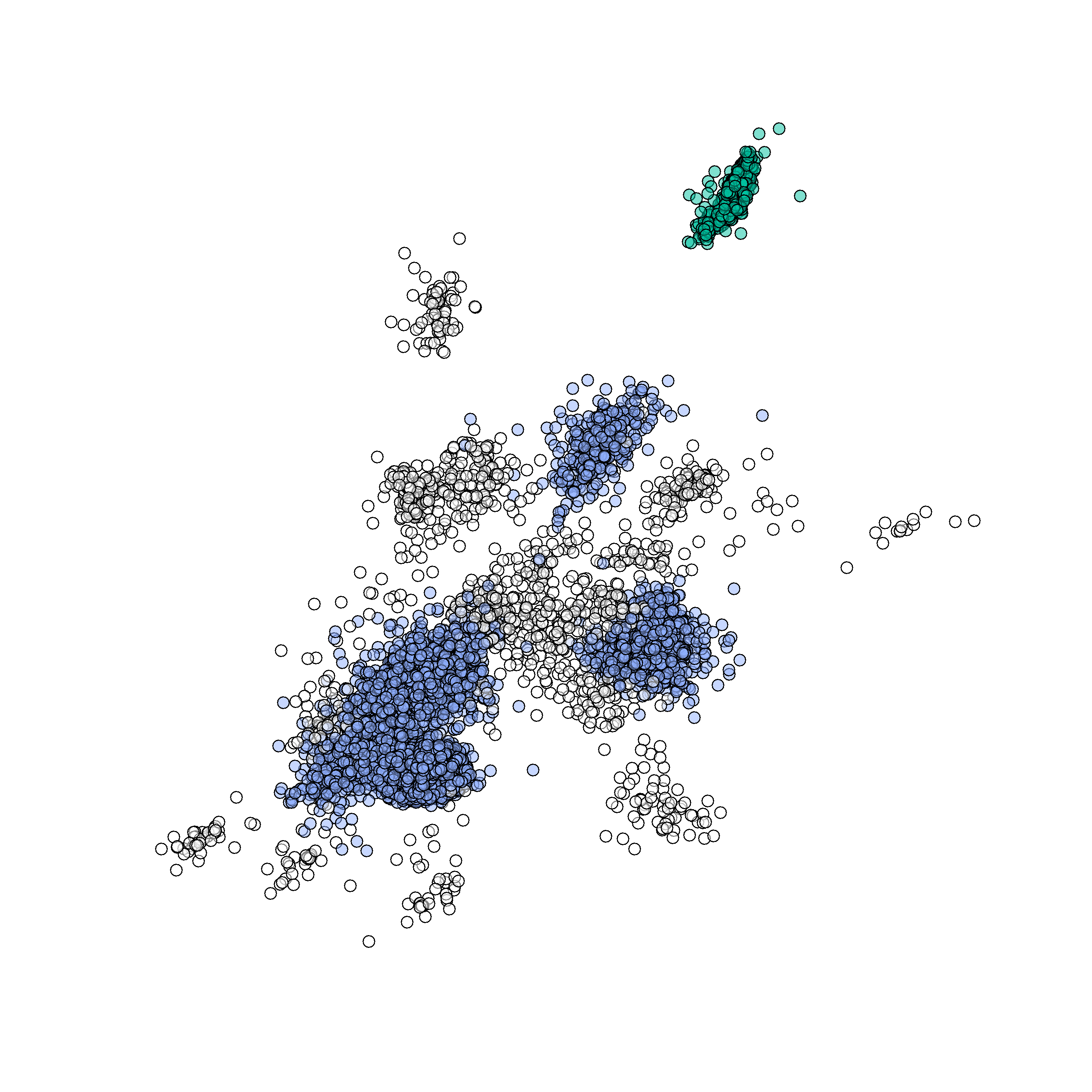

Supplement: S3 Fig — (PNG) [file pone.0305583.s004.png]

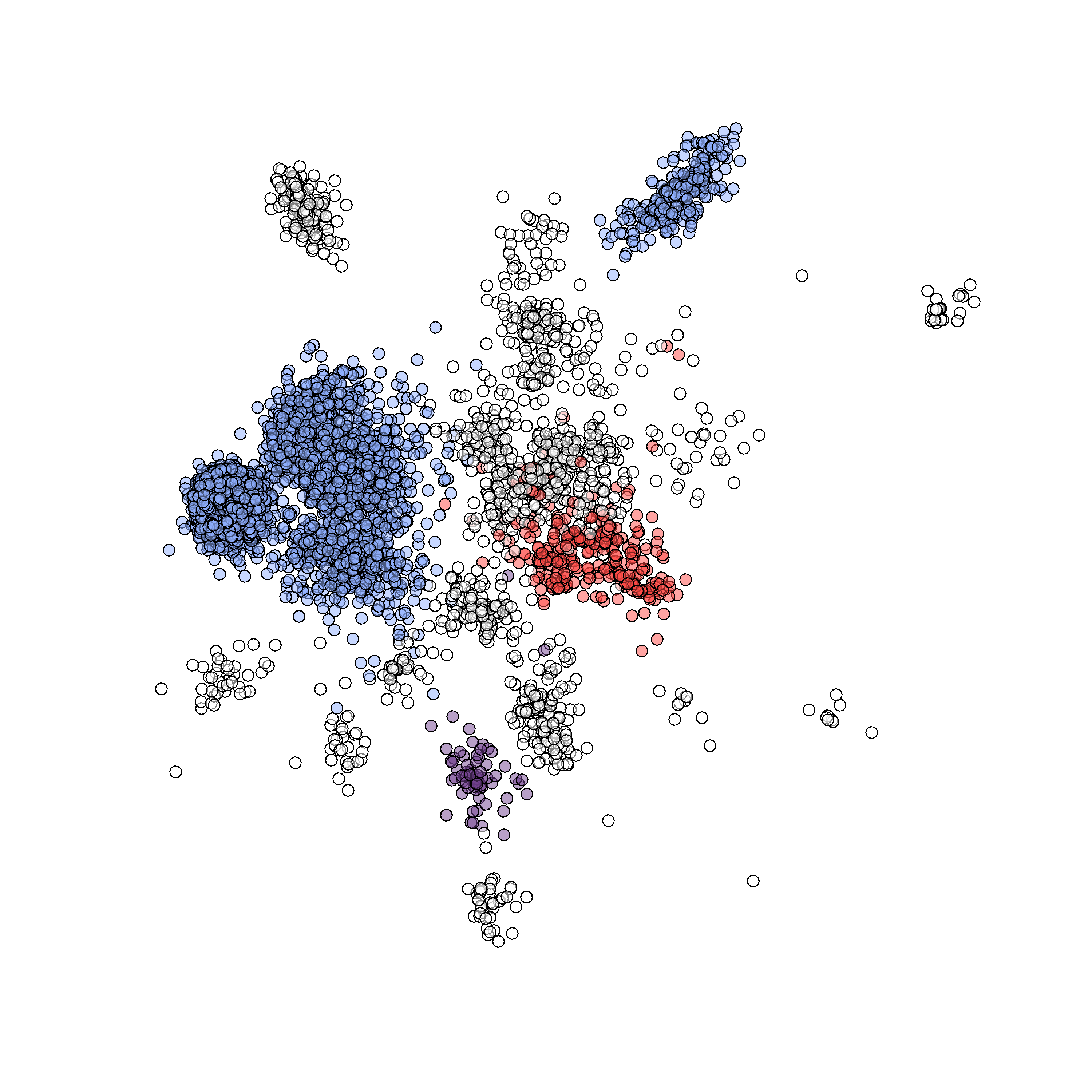

Supplement: S4 Fig — (PNG) [file pone.0305583.s005.png]

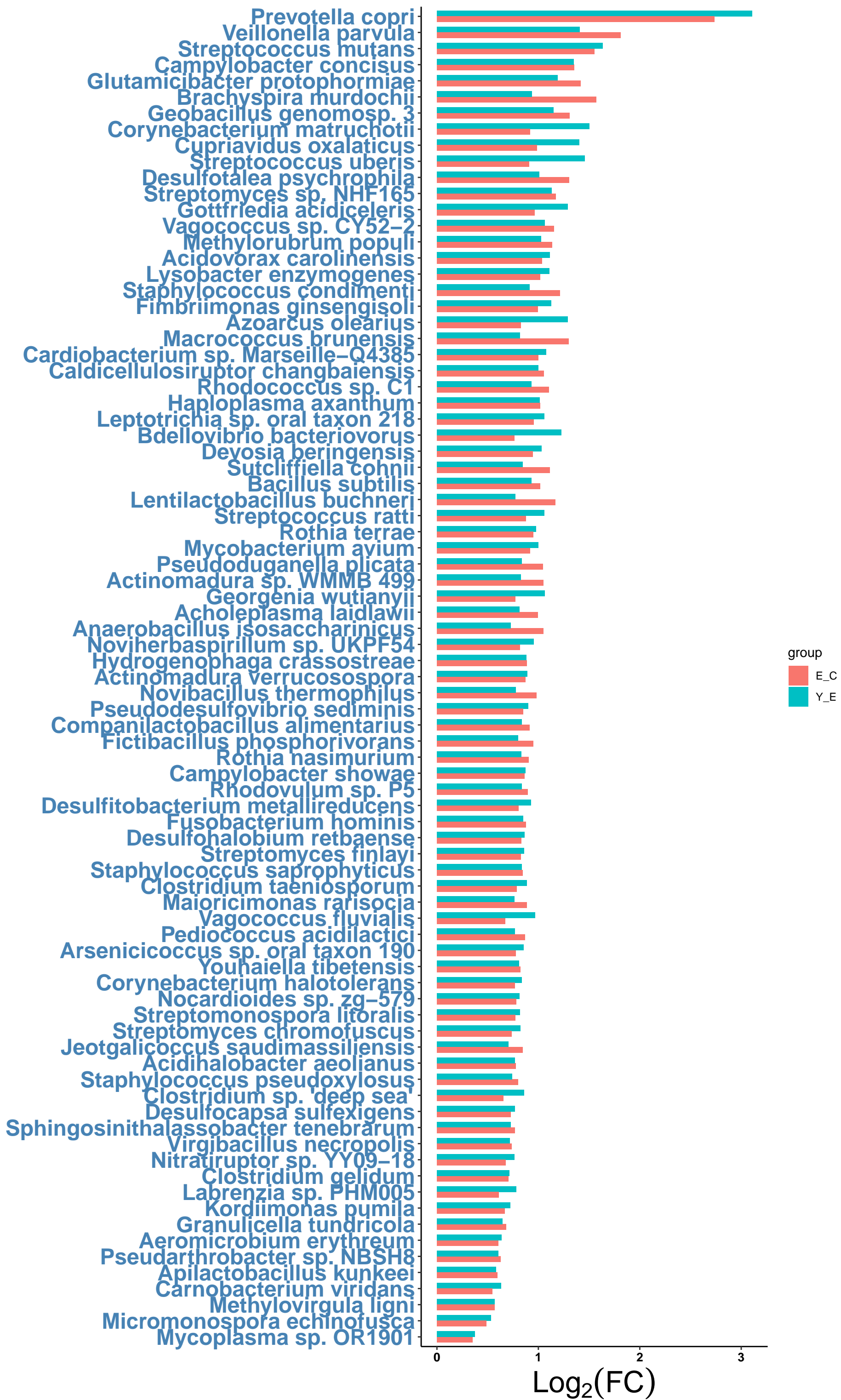

Supplement: S5 Fig — (PDF) [file pone.0305583.s006.pdf]

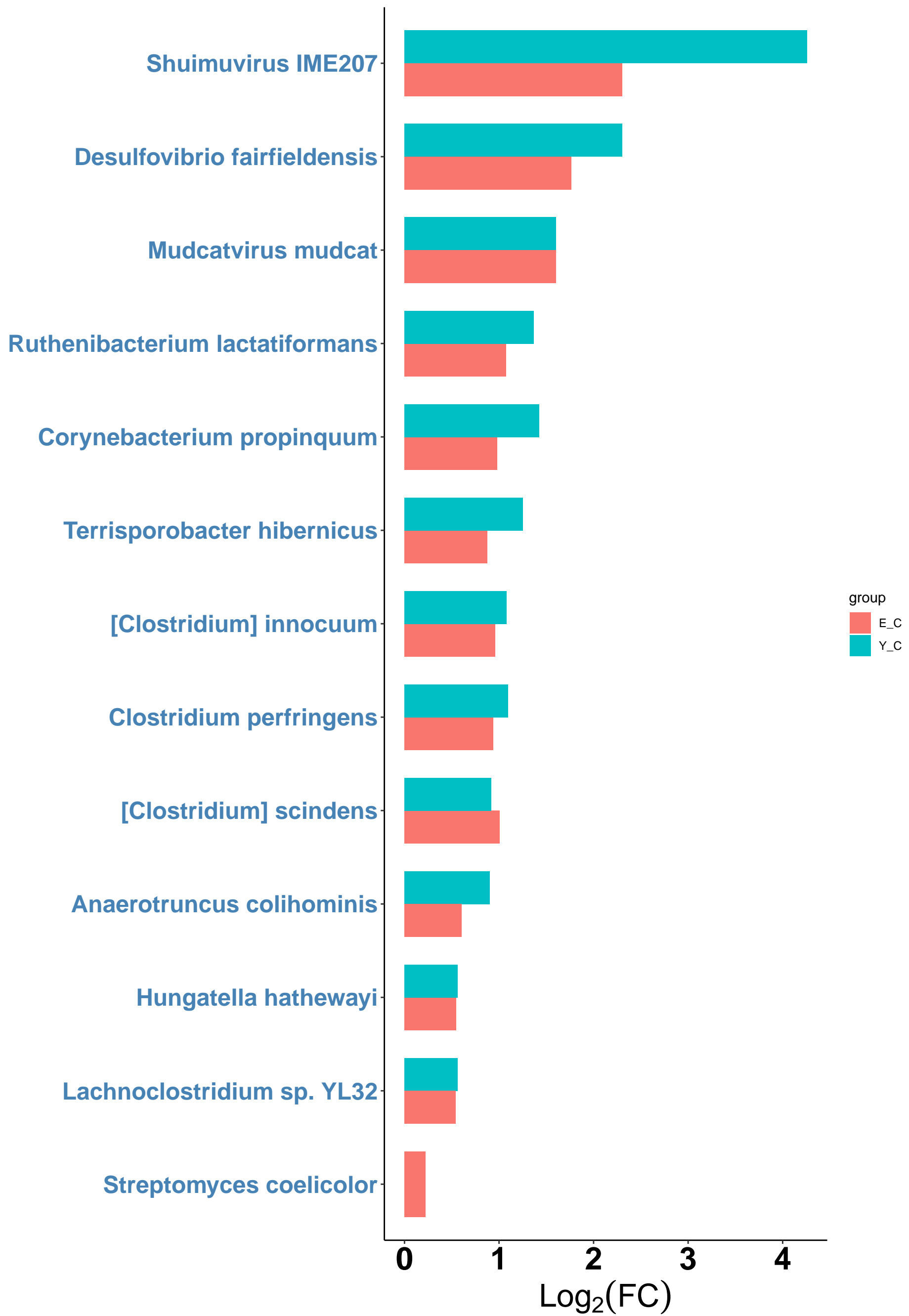

Supplement: S6 Fig — (PDF) [file pone.0305583.s007.pdf]

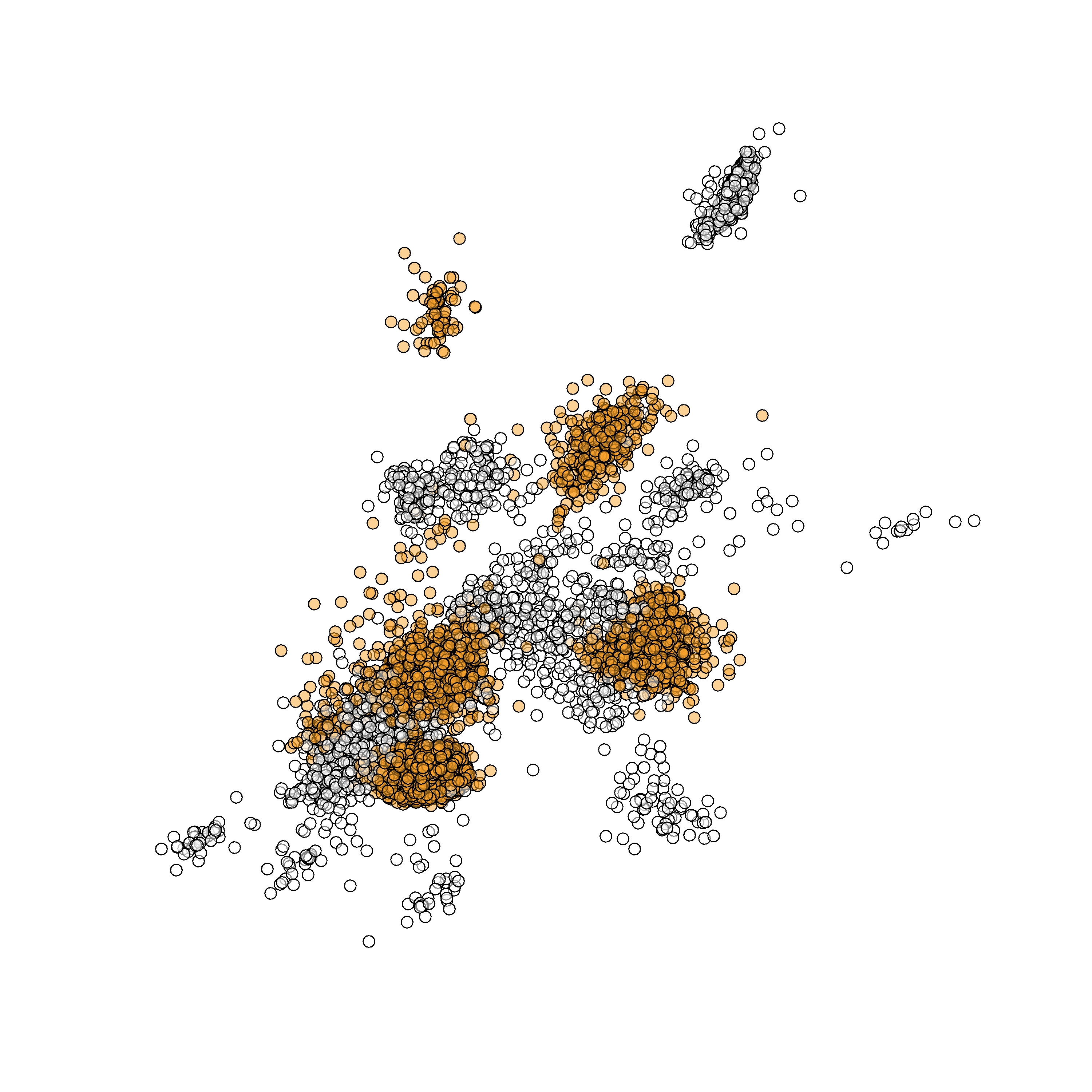

Supplement: S7 Fig — (PNG) [file pone.0305583.s008.png]

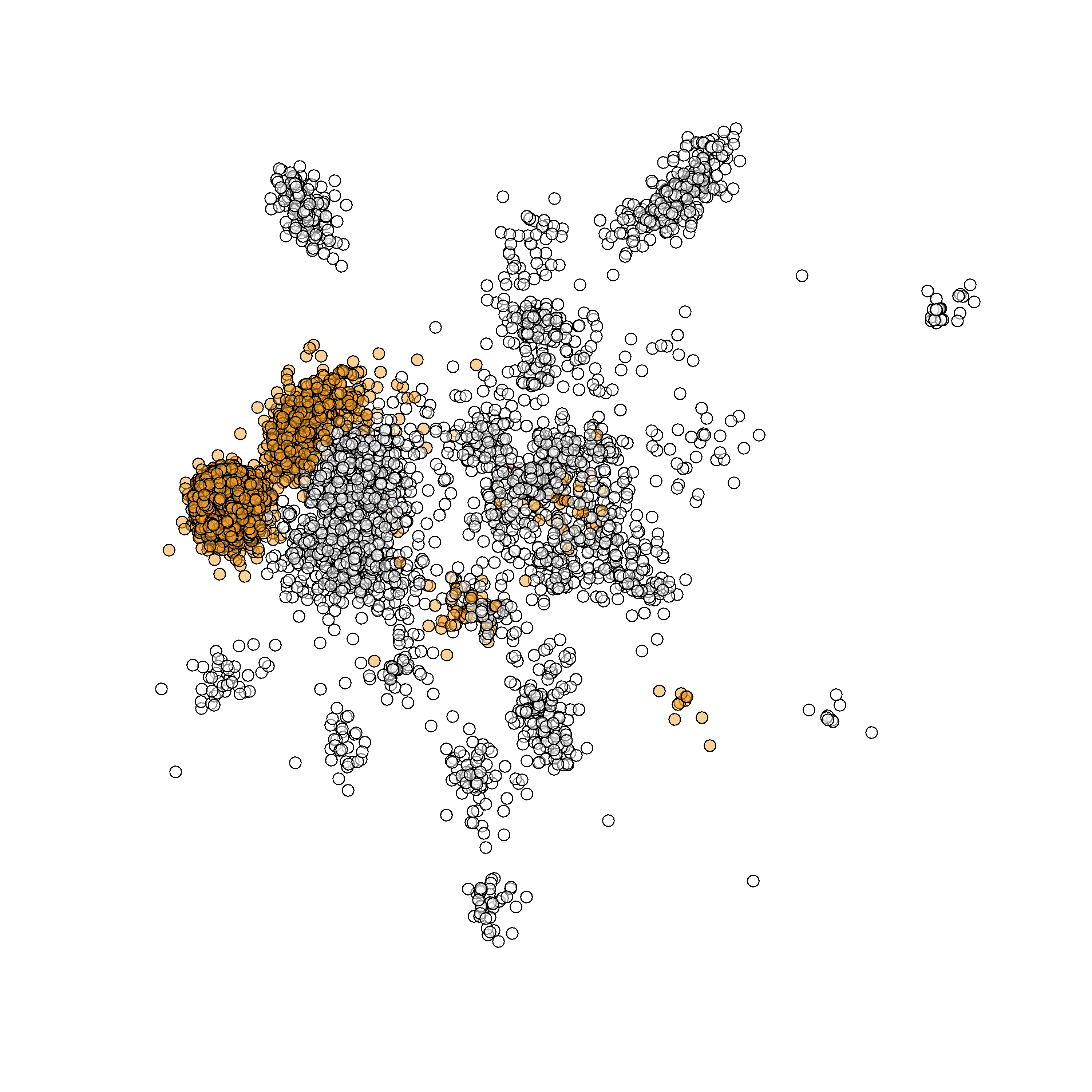

Supplement: S8 Fig — (PNG) [file pone.0305583.s009.png]

Prevotella copri interactions in E clusters

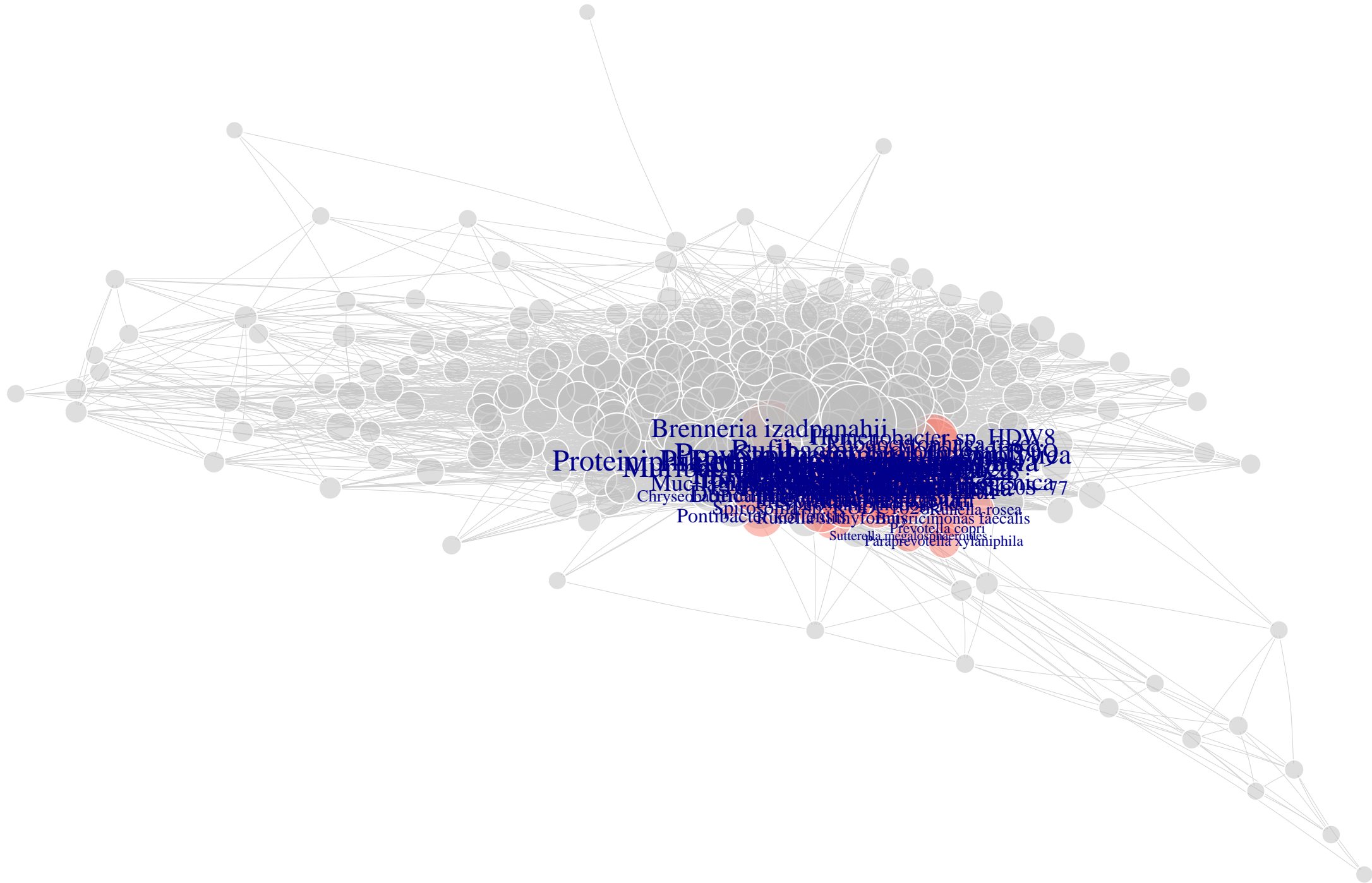

Supplement: S9 Fig — (PDF) [file pone.0305583.s010.pdf]

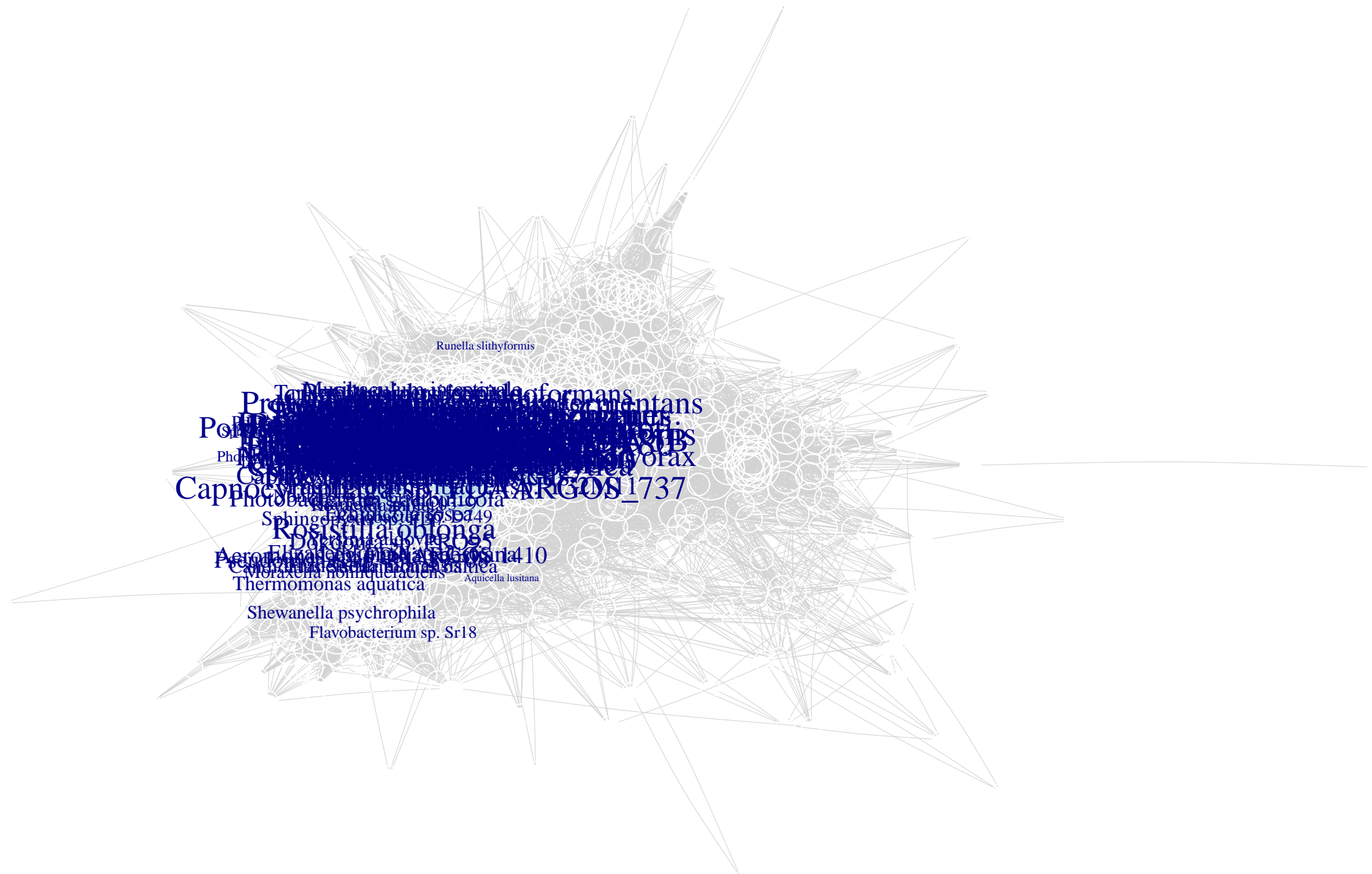

Supplement: S10 Fig — (PDF) [file pone.0305583.s011.pdf]

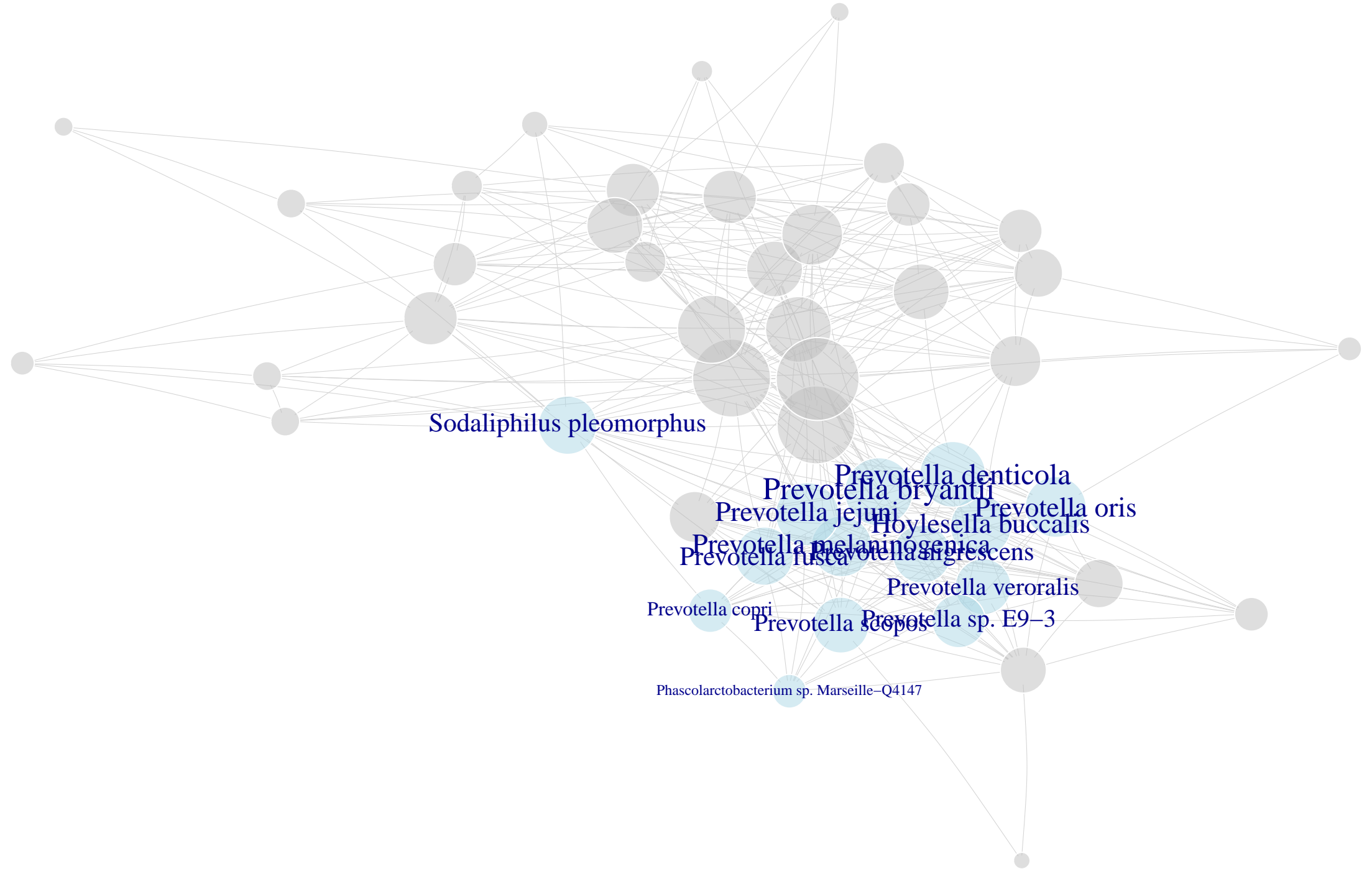

Supplement: S11 Fig — (PDF) [file pone.0305583.s012.pdf]

### Shuimuvirus IME207 interactions in C cluster

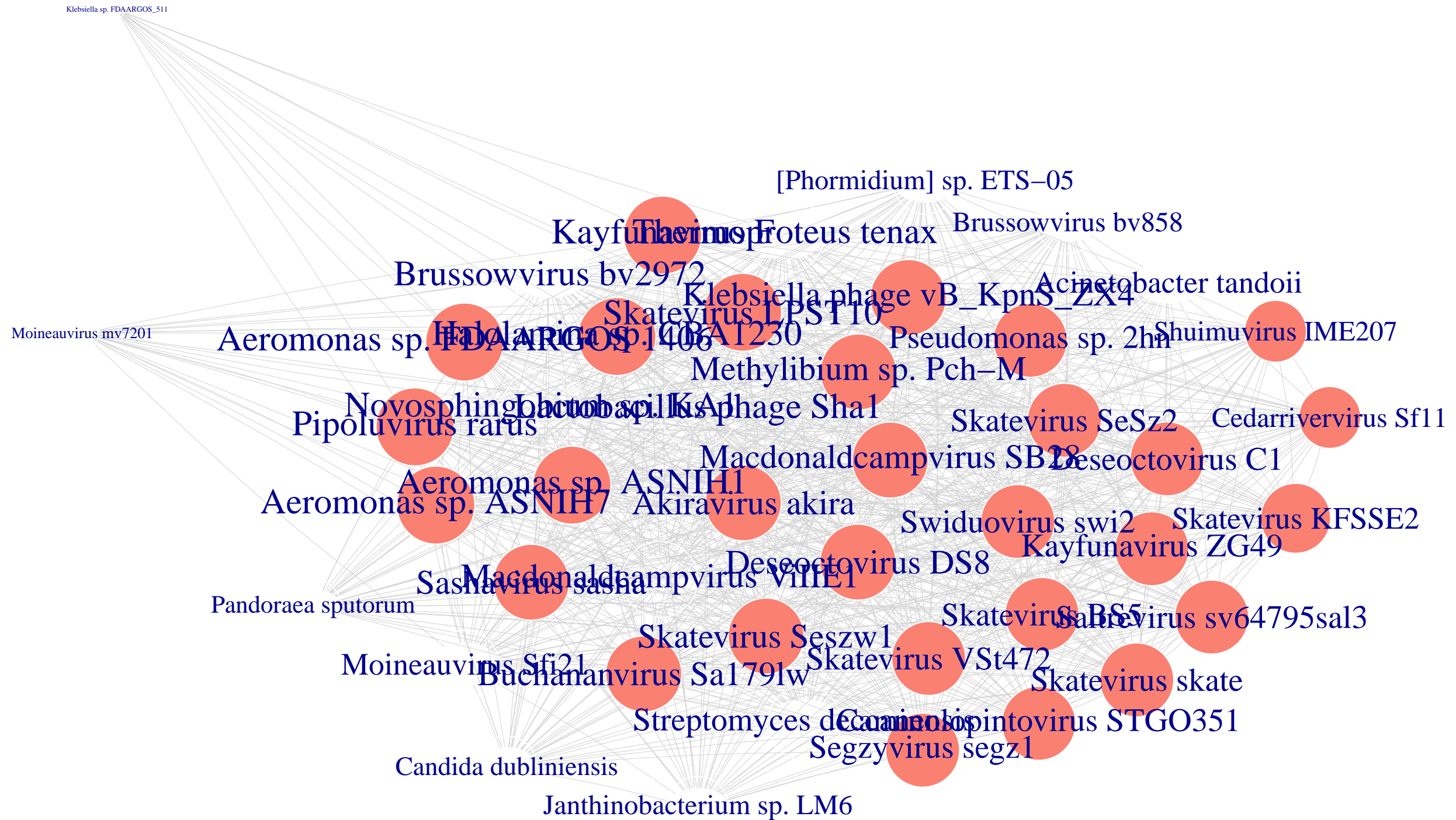

Supplement: S12 Fig — (PDF) [file pone.0305583.s013.pdf]

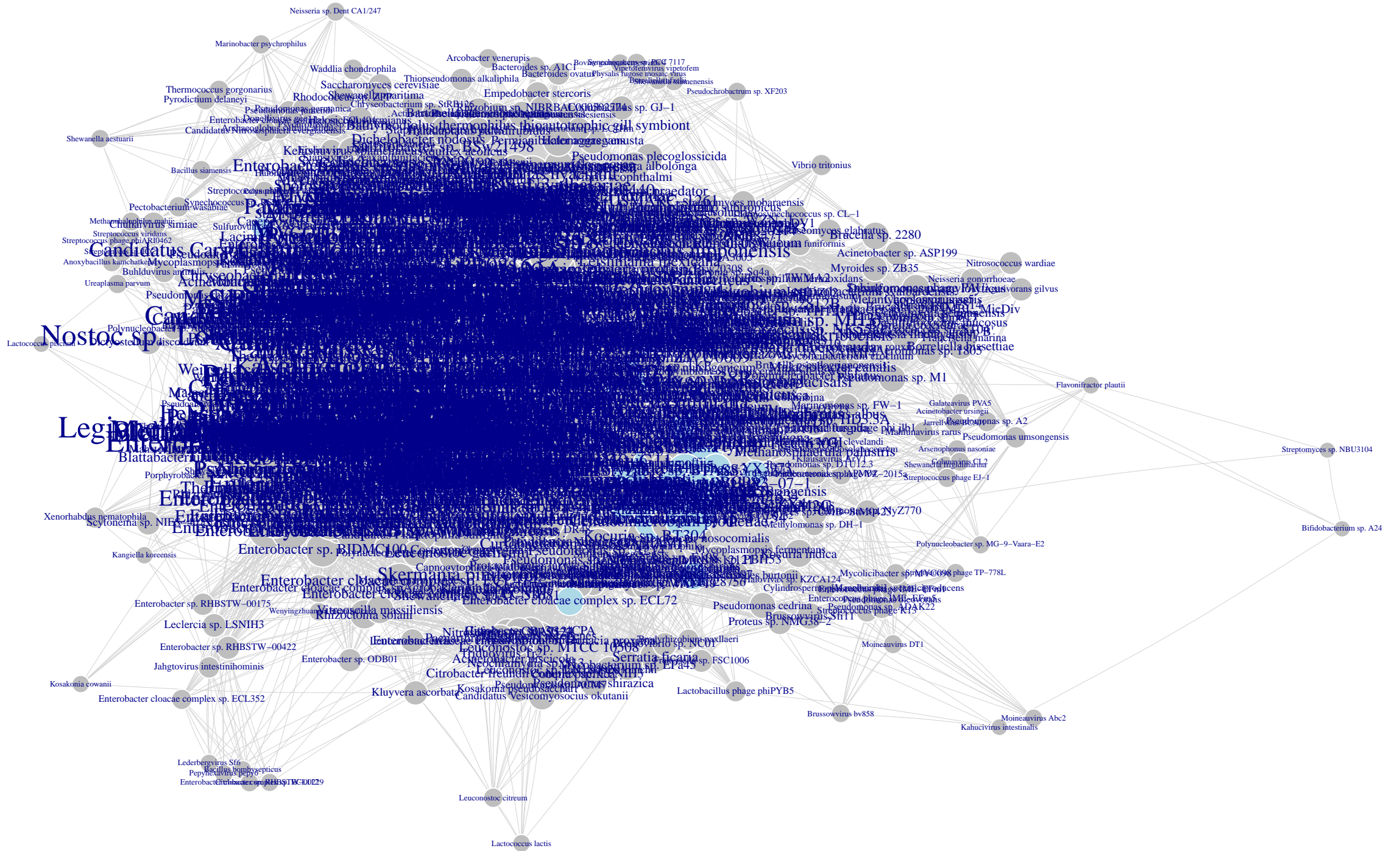

Supplement: S13 Fig — (PDF) [file pone.0305583.s014.pdf]
